# Cyclo-*para*-azulenes: Segments of Metallic Nonalternant Carbon Nanotubes

**DOI:** 10.1021/jacs.6c05318

**Published:** 2026-05-20

**Authors:** Yan Wang, Jian Sun, Xiaohe Miao, Junzhi Liu

**Affiliations:** † Department of Chemistry, 25809 The University of Hong Kong, Pokfulam Road, Hong Kong 999077, P.R. China; Γ Chemistry and Chemical Engineering of Guangdong Laboratory, Shantou 515031, P.R. China; ⊥ Instrumentation and Service Center for Physical Sciences, 557712 Westlake University, Hangzhou, Zhejiang 310024, P.R. China; § State Key Laboratory of Synthetic Chemistry, HKU-CAS Joint Laboratory on New Materials and Shanghai-Hong Kong Joint Laboratory on Chemical Synthesis, 25809 The University of Hong Kong, Pokfulam Road, Hong Kong 999077, P.R. China; # Materials Innovation Institute for Life Sciences and Energy (MILES), HKU-SIRI, Shenzhen 518045, P.R. China

## Abstract

Since the first discovery in 1991, carbon nanotubes (CNTs) have been widely studied, yet, the precise synthesis of structurally defined CNTs remains challenging. [*n*]­Cyclo-*para*-phenylenes (CPPs), segments of CNTs, have been explored as potential precursors for the synthesis of CNTs. While nonalternant distortions are theoretically predicted to tune the electronic properties and yield metallic CNTs, such structures have remained elusive. Herein, we report the first synthesis of the fully nonalternant [8]- and [10]­cyclo-*para*-azulenes (**[8]­CPA** and **[10]­CPA**) via a platinum-mediated catalyst, which can be considered as the segments of metallic nonalternant carbon nanotubes. Single-crystal X-ray diffraction analysis confirms the fully nonalternant cyclic framework of **[8]­CPA**. Remarkably, this nanoring exhibits rare straight tubular stacking, forming a supramolecular nonalternant nanotube. **[8]­CPA** and **[10]­CPA** display exceptionally small HOMO–LUMO gaps, smaller even than that of **[4]­CPP** (the smallest and highly strained benzenoid nanoring), validating theoretical predictions that nonalternant topologies reduce the energy gap. The successful synthesis of CPA nanorings with narrowed energy gaps demonstrates a key step toward the realization of metallic nonalternant CNTs.

## Graphical abstract

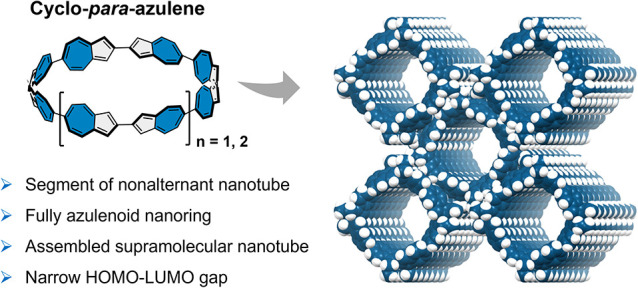

As representative one-dimensional (1D) carbon allotrope, carbon nanotubes (CNTs) have attracted significant research interest since their first discovery by Iijima in 1991 ^1^ (Figure 1a). The cylindrical CNTs display extraordinary properties,^2–8^ enabling diverse applications in chemical and biological sensors,^9–12^ energy devices,^13–15^ supercapacitors,^16^ fiber sheets^17,18^ and so on. Despite the fact that CNTs can be successfully produced,^19–22^ they are typically obtained as mixtures with varied diameters or edge structures which are limited by the separation processes.^22–25^ Achieving precise control over uniform size and specific edge geometry remains a formidable challenge until now. Thus, in order to elucidate the structure–property relationship of CNT, cyclo-*para*-phenylenes (CPPs) have emerged as ideal segmental models.^26^ In 2008, the first series of [*n*]­CPPs with tunable ring sizes were synthesized by Jasti and co-workers (Figure 1c),^27^ establishing a bottom-up strategy for the rational construction toward uniform CNTs.^28,29^ Fascinated by their unique curved, strained frameworks and intriguing π-conjugated systems, Jasti,^27,30–35^ Itami,^36–39^ Yamago,^40–44^ Isobe^45^ and other groups^46–50^ subsequently synthesized various [*n*]­CPPs (*n* = 5–16, 18) within one decade. With the gradually matured synthesis methodologies, series of CPP derivatives have been developed consequently,^51–64^ including CaNAP as shown in Figure 1c.^65,66^ A milestone in CNT construction came in 2013, when Itami and co-workers successfully synthesized uniform CNTs via chemical vapor deposition (CVD) using CPPs as seed templates.^67^ This work confirmed the viability of bottom-up, segment-based strategy, and underscored the importance of well-defined molecular segments with structural precision.

**Figure 1:**
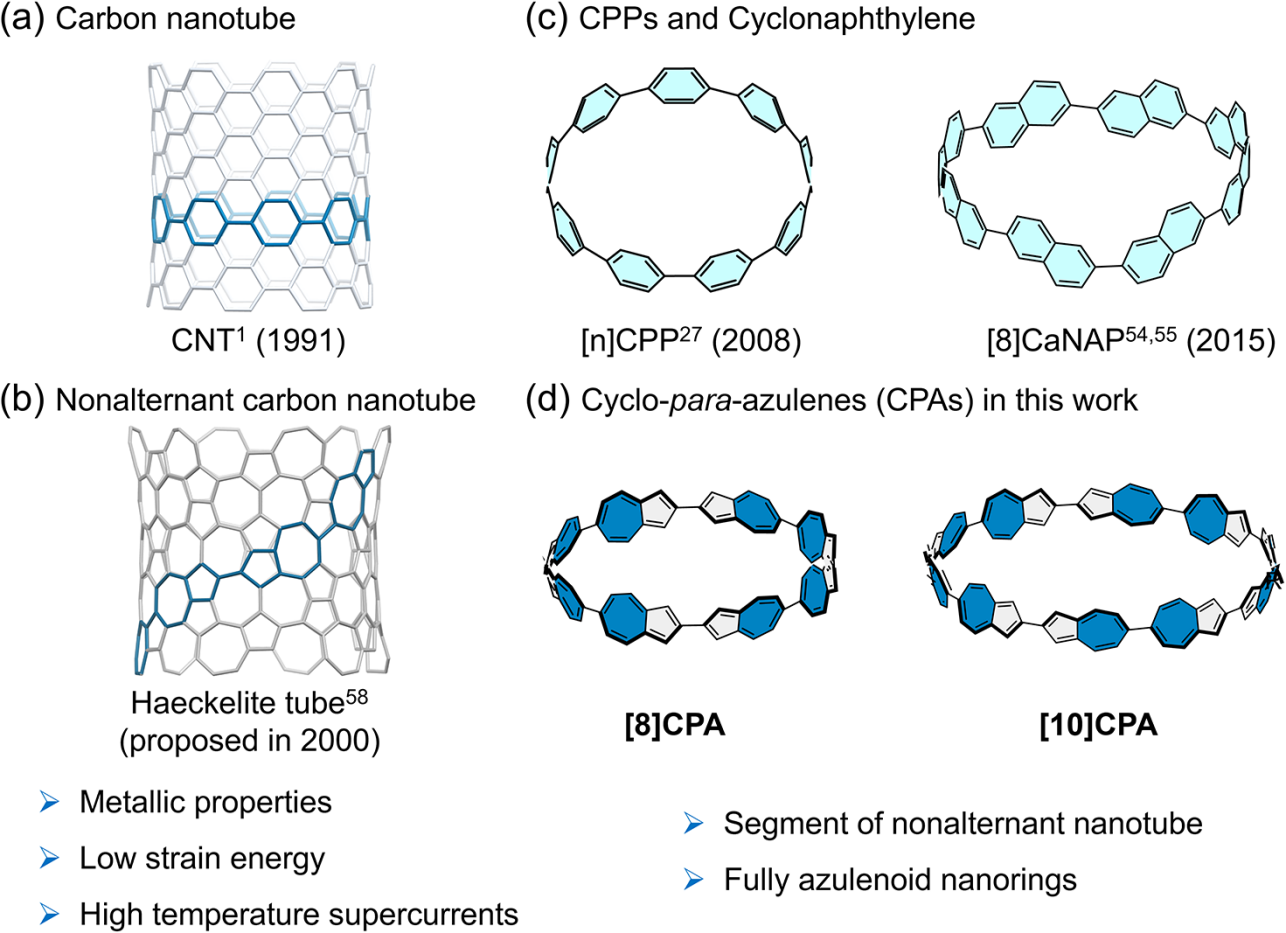
(a) Carbon nanotube first discovered in 1991. (b) Nonalternant carbon nanotube (Haeckelite tube) proposed in 2000. (c) Representative CPPs and cyclonaphthylenes. (d) Fully nonalternant cyclo-*para*-azulene (CPAs) synthesized in this work.

Besides the sizes and edge structures, intrinsic topological transformation was also predicted as a key impact on the electronic properties of carbon allotropes. With the metallic properties and high density of state predicted for pentaheptite and haeckelite featuring nonalternant distortions (pentagons and heptagons) as inspiration,^68,69^ 1D counterparts, nonalternant CNTs, were also predicted theoretically. In 2000, Terrones et al. have proposed novel Haeckelite nanotubes^69^ composed of ordered pentagons and heptagons (R5,7 (6, 0) tube in Figure 1b). Calculations predicted that these Haeckelite tubes would exhibit low stain energy, superconductivity, high temperature supercurrents and metallic properties. As a result, these tubes are considered to have great potential in field emitting devices owing to the closed band gap.^70,71^ Despite the fact that Lambin and Biró have proposed that the Haeckelite nanotubes could be formed by directly rolling azulene units (consisting of fused pentagon-heptagon pairs),^72^ and Haeckelite-like patterns have been observed in various morphologies through microscopy,^73–75^ the precise synthesis of nonalternant CNTs remains challenging. In contrast to their benzenoid counterparts, even construction of a segmental template, such as an azulene-based nanoring, has yet to be reported.

Herein, we designed cyclo-*para*-phenylene analogs incorporating only azulene units, termed cyclo-*para*-azulenes (CPAs, Figure 1d), as the shortest nonalternant CNT segments. To date, the only example of azulene incorporation into one cyclic nanoring was reported by Jiang and co-workers in 2023,^76^ demonstrating the substantial challenge of embedding nonalternant units into strained cyclic nanorings. In this work, we successfully synthesize fully nonalternant [8]- and [10]­cyclo-*para*-azulenes (**[8]­CPA** and **[10]­CPA**) (Figure 1 d). The structure of **[8]­CPA** with ethyl groups is confirmed by an X-ray single-crystal analysis, revealing a rare straight tubular packing arrangement. As a result, it is assembled as supramolecular nonalternant nanotubes. UV–vis spectroscopy and theoretical calculations show that both CPAs exhibit narrower energy gaps (Δ*E*_g_^cal^ = 2.28 eV for **[8]­CPA**) compared to benzenoid CPPs, surpassing even that of the highly strained **[4]­CPP** (Δ*E*_g_^cal^ = 2.50 eV, the smallest benzenoid nanoring), highlighting the electronic impact of the entire nonalternant topology.

We selected compound **6** (Scheme 1) as the precursor for the construction of CPA nanorings. The introduction of an ethyl group increases the solubility of the diazulene units, which is vital for subsequent cyclization and purification. The overall synthetic route toward **[8]­CPA** and **[10]­CPA** is presented in Scheme 1. Compound **1** was obtained through the Sandmeyer reaction (confirmed via a single crystal analysis shown in Figure S23). Then ester groups were removed smoothly under strong acid conditions. After obtaining **2**, an ethyl group was introduced at the 1-position of azulene via a Friedel-Crafts acylation followed by reduction. The chlorine atom at the 6-position was successfully transformed into a boronic ester. Then, the key precursor **6** was subsequently synthesized via the Yamamoto reaction in high yield. After the final cyclization and elimination, two nanorings, **[8]­CPA** and **[10]­CPA**, were successfully isolated. These separated nanorings represent the first example of cyclic structures composed of entirely pentagon-heptagon pairs.

**Scheme 1:**
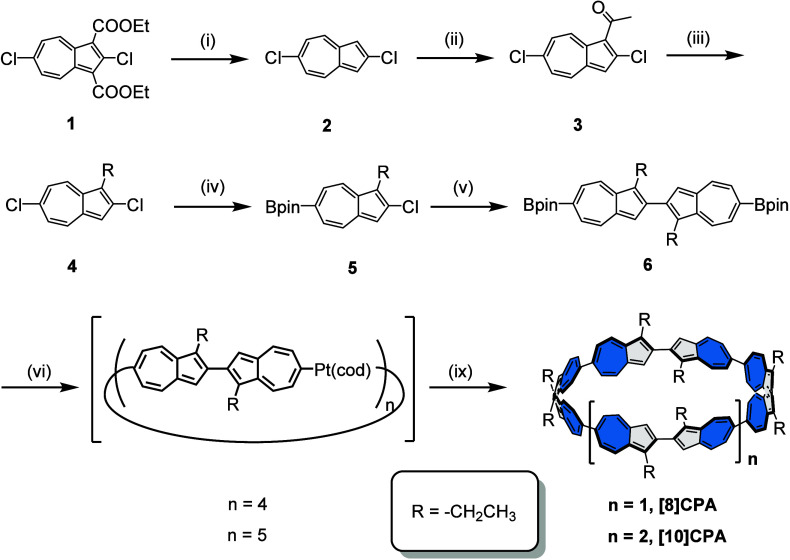
Synthetic Route towards **[8]­CPA** and **[10]­CPA** ^a^

a Conditions: (i) H_3_PO_4_, H_2_SO_4_, 90 °C, 30 min, 76.9%; (ii) ethanoyl chloride, AlCl_3_, DCM, 0 °C, 5 h, 43.8%; (iii) boron trifluoride-diethyl etherate complex, borane-tetrahydrofuran complex, THF, 0 °C, 30 min, 82.0%; (iv) bis­(pinacolato)­diboron, Pd­(dppf)­Cl_2_, KOAc, 1,4-dioxane, 60 °C, 12 h, 64.4%; (v) Ni­(cod)_2_, bpy, cod, DMF, 110 °C, 24 h, 52.2%; (vi) Pt­(cod)­Cl_2_, CsF, THF, 70 °C, 24 h; (ix) PPh_3_, toluene, 100 °C, 24 h. **[8]­CPA**: 1.8%, **[10]­CPA**: <1.0%.

The structures of **[8]­CPA** and **[10]­CPA** were confirmed by ^1^H NMR and high-resolution MS. The well-resolved ^1^H NMR spectra of **[8]­CPA** and **[10]­CPA** reveal five aromatic signals for each compound (Figure 2a), consistent with their similar skeletons. Owing to its highly symmetrical structure, **[8]­CPA** displays four doublets peaks (8.3, 7.9, 7.5, and 6.9 ppm, labeled as H^2^, H^5^, H^3^, and H^4^) and one singlet peak (7.1 ppm, labeled as H^1^) with equal integration, while **[10]­CPA** exhibits its five signals at 8.4, 8.1, 7.5, 7.1, and 7.3 ppm, respectively. The general downfield shift observed for **[10]­CPA** compared with **[8]­CPA** is attributed to its increased size, mirroring the behavior of cyclo-*para*-phenylenes.^77^ Variable-temperature ^1^H NMR spectra of the CPAs (Figures S17–S20) reveal their dynamic behavior. In addition, the substituted ethyl groups in each azulene unit are chemically equivalent, demonstrating that the nanorings undergo free rotation in solution at room temperature. Moreover, MALDI-TOF MS analysis confirmed the molecular weight (Figures 2b and 2c), with observed values (*m*/*z*) of 1232.6217 and 1541.7890 for **[8]­CPA** and **[10]­CPA**, respectively. The excellent match between the experimental and simulated isotopic patterns (insets) provides further evidence for the successful synthesis of target CPA nanorings.

**Figure 2:**
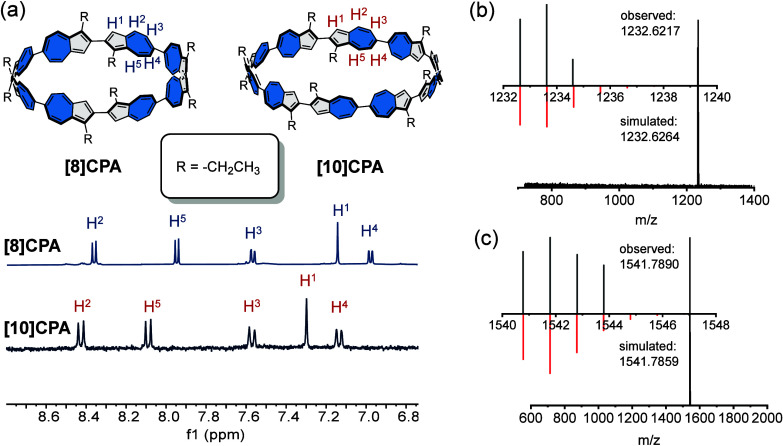
(a) ^1^H NMR spectra of **[8]­CPA** and **[10]­CPA**. MALDI-TOF MS spectra of (b) **[8]­CPA** and (c) **[10]­CPA**, respectively. The inset indicates the observed signal values (black) and simulated signal values (red).

Needle-type single crystals of **[8]­CPA** suitable for X-ray diffraction analysis were obtained via diffusion in DCM/CS_2_. Despite many attempts under various conditions, high-quality single crystals of **[10]­CPA** could not be obtained. From the top and side views (Figures 3a and 3b), **[8]­CPA** demonstrates a closed cylindrical shape. A well-defined cavity could also be observed with a diameter of 17.5 Å, comparable to that of [12]­CPP.^36^ The two **[8]­CPA** nanorings in the unit cell, which possess low *C*_4_ symmetry, are connected only through the van der Waals intermolecular C–H···C interactions (London dispersion force)^78,79^ with a distance of 2.889 Å between C and H (Figure 3c). These van der Waals interactions were further visualized via a noncovalent interaction (NCI) plot, shown as green isosurfaces (Figure S25). No typical π-π interactions are present within the nanoring system of **[8]­CPA**. In contrast to the compact herringbone packing commonly observed in traditional CPP crystals, **[8]­CPA** crystallizes a rare straight tubular packing arrangement with an interlayer distance of 6.34 Å (Figure 3d, Figure S24). Each **[8]­CPA** ring stacks on top of the other, assembling one-dimensional ordered supramolecular nanotubes, and the distance between the centers of two adjacent **[8]­CPA** is 14.155 Å (Figure 3e). The total strain energies of **[8]­CPA** and **[10]­CPA** are analyzed to be 46.5 and 38.7 kcal/mol via strainviz, which are slightly lower than those of the isomers **[8]­CaNAP** and **[10]­CaNAP** (Figure S26). Most of the strain originates from the dihedral part between azulene units, while bond and angle strain contribute only minimally (Table S3). Additionally, both CPAs display small total dipole moments (Figure S27, Table S4).

**Figure 3:**
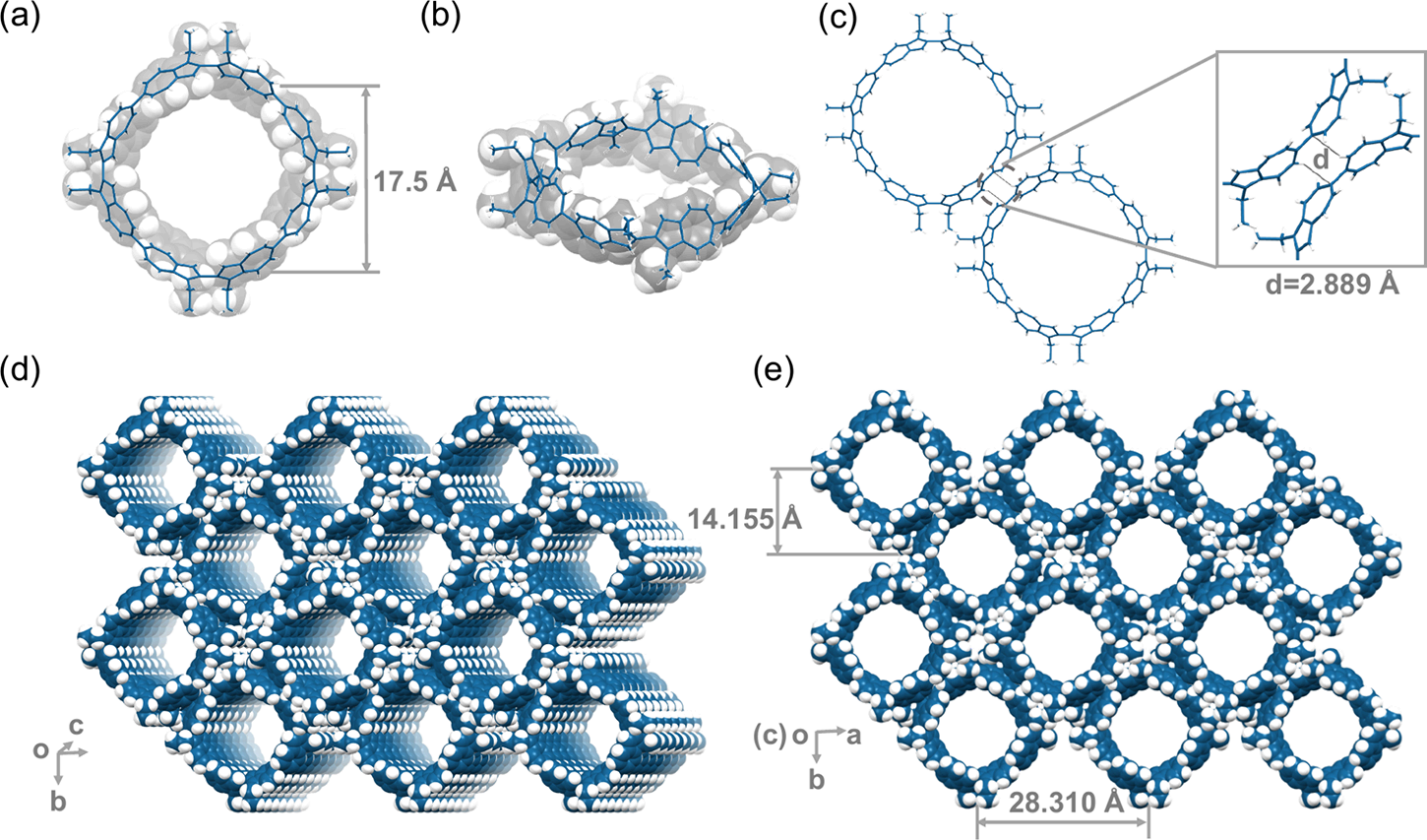
Single-crystal analysis of [**8]­CPA**. (a, b) Top and side views of **[8]­CPA**. (c) Repeating unit of **[8]­CPA** in one unit cell from the top view. (d, e) Side and top views of the packing structure of **[8]­CPA**.

The UV–vis absorption spectra of **[8]­CPA** and **[10]­CPA** were measured in DCM as shown in Figure 4a. Both spectra exhibit two distinct absorption peaks, demonstrating a size-insensitivity trend with slight shifts. The two major absorption peaks of **[8]­CPA** located at 340 and 478 nm, while those for **[10]­CPA** appear at 330 and 480 nm. Notably, the absorption tails extend into the near-infrared region (NIR-I), which can be attributed to the intrinsic properties of the azulene unit.^80–83^ The optical energy gaps of **[8]­CPA** and **[10]­CPA** are calculated to be 1.89 and 1.91 eV (Figure S30), presenting a trend similar to that of the optical bandgap obtained from the UV–vis spectra (Figure S34). Additionally, the fluorescence spectrum of **[8]­CPA** exhibits a low-intensity emission band with a maximum peak at 518 nm. Upon ring expansion, the emission maximum of **[10]­CPA** shifts slightly to 510 nm, showing a minor blue shift. Additionally, both CPAs demonstrate solvent-dependent charge transfer behavior, arising from the intrinsic properties of the core azulene units (Figure S33). To elucidate the electronic structure and orbital distribution introduced by incorporating azulene units into the nanoring framework, DFT calculations were performed on the CPAs at the B3LYP/6-31g­(d) level (Figure 4d).^84,85^ For **[8]­CPA**, the calculated HOMO and LUMO energies are – 5.32 and 3.04 eV, respectively, corresponding to an energy gap of 2.28 eV. **[10]­CPA** exhibits similar values (−5.34 and 3.02 eV), yielding a slightly wider gap of 2.32 eV. As the number of azulene units increases, the HOMO–LUMO gap slightly widens, and both nanorings display delocalized frontier molecular orbitals. Compared to their benzenoid isomers naphthalene nanorings (**[8]­CaNAP** and **[10]­CaNAP**, Figure 4d), the HOMO and LUMO levels of CPAs both decreased, resulting in a significantly reduced energy gap, by more than 0.9 eV. Notably, the energy gap of CPAs is even narrower than that of the highly strained **[5]­CPP** (Δ*E*_g_^cal^ = 2.71 eV)^43^ and [**4]­CPP** (Δ*E*_g_^cal^ = 2.50 eV)^42^ (Figure 4c), underscoring the profound impact of azulenes incorporation on modulating the nanoring energy gap. To further assess the effect of the cyclic topology, the frontier molecular orbitals of linear counterparts of **[8]­CPA** and **[10]­CPA** were also calculated. In contrast to the delocalized orbitals of the cyclic CPAs, the linear molecules show apparent charge separation and slightly wider energy gap. Cyclic voltammetry (CV) measurements of both CPAs were performed in DCM under nitrogen atmosphere (Figures S31 and S32). **[8]­CPA** and **[10]­CPA** display obvious reduction potentials at −1.02 and −1.05 V, respectively. Moreover, the nucleus-independent chemical shifts (NICS) calculations were performed (Figures S28 and S29). Although the NICS values are reduced compared to typical aromatic systems, the CPAs retain aromatic features. Based on these calculation insights into their photophysical properties, the cyclic CPAs hold considerable promise for applications in electrical devices.

**Figure 4:**
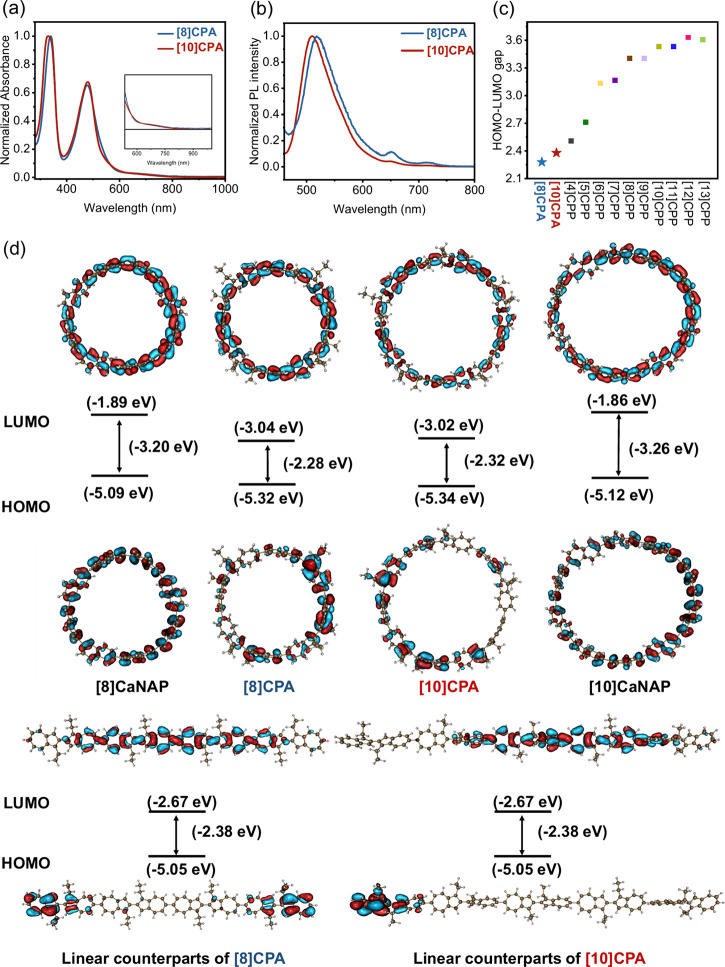
(a) Normalized UV–vis absorption spectra of **[8]­CPA** and **[10]­CPA** in DCM. The inset shows the enlarged absorption in the range of 535–1000 nm. (b) Normalized fluorescence spectra of **[8]­CPA** and **[10]­CPA** in DCM. (c) Calculated HOMO–LUMO gap of **[8]­CPA**, **[10]­CPA** and [*n*]­CPPs (*n* = 4–13). (d) Comparison of DFT-calculated frontier molecular orbitals of **[8]­CPA** and **[10]­CPA**, their corresponding linear counterparts and benzenoid [8]­CaNAP and [10]­CaNAP at B3LYP/6-31g­(d) level.

In summary, we have achieved the first synthesis of fully nonalternant carbon nanorings, **[8]­CPA** and **[10]­CPA**. Their structures were confirmed by NMR and high-resolution MS, and for [**8]­CPA** by X-ray crystallography. In addition, the revealed unusual straight tubular packing leads to assembled supramolecular nonalternant nanotubes. Moreover, the CPAs exhibit size-independent absorption behavior and a slight blue-shift in emission due to the enlarged size. Most importantly, theoretical calculations demonstrated that these nanorings possess exceptionally narrow energy gaps (Δ*E*_g_^cal^ = 2.28 eV for [**8]­CPA**; Δ*E*_g_^cal^ = 2.50 eV for [**4]­CPP**), placing them among the smallest reported for carbon-based nanorings. The successful synthesis of CPAs with reduced energy gaps represents a significant step toward the development of metallic nonalternant CNTs and their potential application in electrical devices.

## Supplementary Material



## Notes

Experimental section, NMR spectra, high-resolution mass spectrometry (HRMS) spectroscopic data, DFT calculations, and additional supporting figures/tables (PDF)
