# Supplementary material for: Cyclo-para-azulenes: Segments of Metallic Nonalternant Carbon Nanotubes
Source: J Am Chem Soc. 2026 May 20;148(21):21238–45. doi: 10.1021/jacs.6c05318 (PMC13244433; doi:10.1021/jacs.6c05318)
Supplement: Supplementary file 1 [file ja6c05318_si_001.pdf]

Supporting Information

Cyclo-*para*-azulenes: Segments of Metallic Nonalternant Carbon Nanotubes

Yan Wang,[†,Γ,‡] Jian Sun,[†,‡] Xiaohe Miao,[⊥] and Junzhi Liu[†,§,#,*]

[†]Department of Chemistry, The University of Hong Kong, Pokfulam Road, Hong Kong 999077, P.R. China.

[Γ]Chemistry and Chemical Engineering of Guangdong Laboratory, Shantou 515031, P.R. China.

[⊥]Instrumentation and Service Center for Physical Sciences, Westlake University, Hangzhou, Zhejiang 310024, P.R. China.

[§]State Key Laboratory of Synthetic Chemistry, HKU-CAS Joint Laboratory on New Materials and Shanghai-Hong Kong Joint Laboratory on Chemical Synthesis, The University of Hong Kong, Pokfulam Road, Hong Kong 999077, P.R. China.

[#]Materials Innovation Institute for Life Sciences and Energy (MILES), HKU-SIRI, Shenzhen 518045, P.R. China.

[‡]These authors contributed equally: Yan Wang & Jian Sun

E-mail: juliu@hku.hk

Table of Contents

1. **General and Materials** 1
2. **Synthesis procedures and NMR spectra characterizations** 2
3. **X-ray crystallography** 20
4. **Theoretical Analysis** 25
5. **CV, UV-vis absorption and fluorescence spectra** 29
6. **High-resolution mass spectrometry** 31
7. **References** 34

1. General and Materials

All the reagents and solvents were purchased to use without any further purification. The precursor **S1** was prepared through the reported literature[1]. The detailed synthesis is described in the following experimental section.

General methods and instruments: All ^{1}H NMR and ^{13}C NMR data were obtained by Bruker 400, 500 or 600 spectrometers (400/500/600 MHz for ^{1}H NMR, 101/126/151MHz for ^{13}C NMR). Data for ^{1}H NMR were presented as following: chemical shifts (δ, ppm), multiplicity (s = singlet, d =doublet, t = triplet, q = quartet, dd = doublet of doublets, tt = triplet of triplets, td= triplet of doublets, m = multiples), coupling constant (Hz), and integration. High resolution mass spectra were performed on a Bruker Q-Tof Maxis II mass spectrometer, DFS high resolution magnetic sector mass spectrometer and MALDI-TOF MS system, Bruker ultrafleXtreme. UV-vis absorption spectra were measured on Agilent Cary 5000, and fluorescence emission spectra were recorded on a Shimadzu RF-530XPC luminescence spectrometer. Cyclic voltammetry measurement was conducted via the three electrode cell on CHI660E (CH Instrument, China) in nitrogen atmosphere with the scan rate of 0.05 V/s. The electrolyte solution was prepared with 0.1M nBu_4NPF_6 and samples in anhydrous DCM. The glass carbon was used as working electrode while a Pt sheet and a silver wire were used as counter and pseudo reference electrode, respectively. All of the potentials were obtained against the ferrocenium/ferrocene redox couple.

2. Synthesis procedures and NMR spectra characterizations

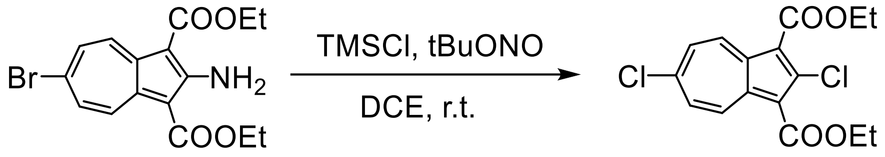

S1 **1**

In a 500 mL round-bottom flask, precursor **S1** (15 g, 40.96 mmol) in the 200 mL 1,2-dichloroethane was bubbled with N_2 for 15 min. In another 250 mL TMSCl (26.18 mL, 204.80 mmol) and tert-Butyl nitrite (t-BuONO) (24.56 mL, 204.80 mmol) in 50 ml 1,2-dichloroethane were prepared with the same procedure simultaneously, and then transferred into **S1** included 500 mL flask under the N_2 atmosphere. The resulting mixture was stirred continuously at room temperature. After 12 h, the solution was quenched with saturated Na_2SO_3 solution and then extracted with DCM with three times. The organic layer was dried with anhydrous Na_2SO_4 and the solvent was evaporated under reduced pressure. The crude product was purified via washing with MeOH for three times to give compound **1** as a red powder (21.50 g, 68 %).

^{1}H NMR (600 MHz, $CDCl_3$): δ 9.34 (d, J = 8.7 Hz, 2H), 7.81 (d, J = 8.8 Hz, 2H), 4.49 (q, J = 7.1 Hz, 4H), 1.47 (t, J = 7.1 Hz, 6H). ^{13}C NMR (151 MHz, $CDCl_3$) δ 164.05, 147.59, 143.55, 139.88, 135.99, 131.19, 116.82, 60.99, 14.49.

HR-MS (ESI) for **1**: calcd. for $C_{16}H_{15}Cl_2O_4$ ([M+H$^+$]): 341.0342, found: 341.0340.

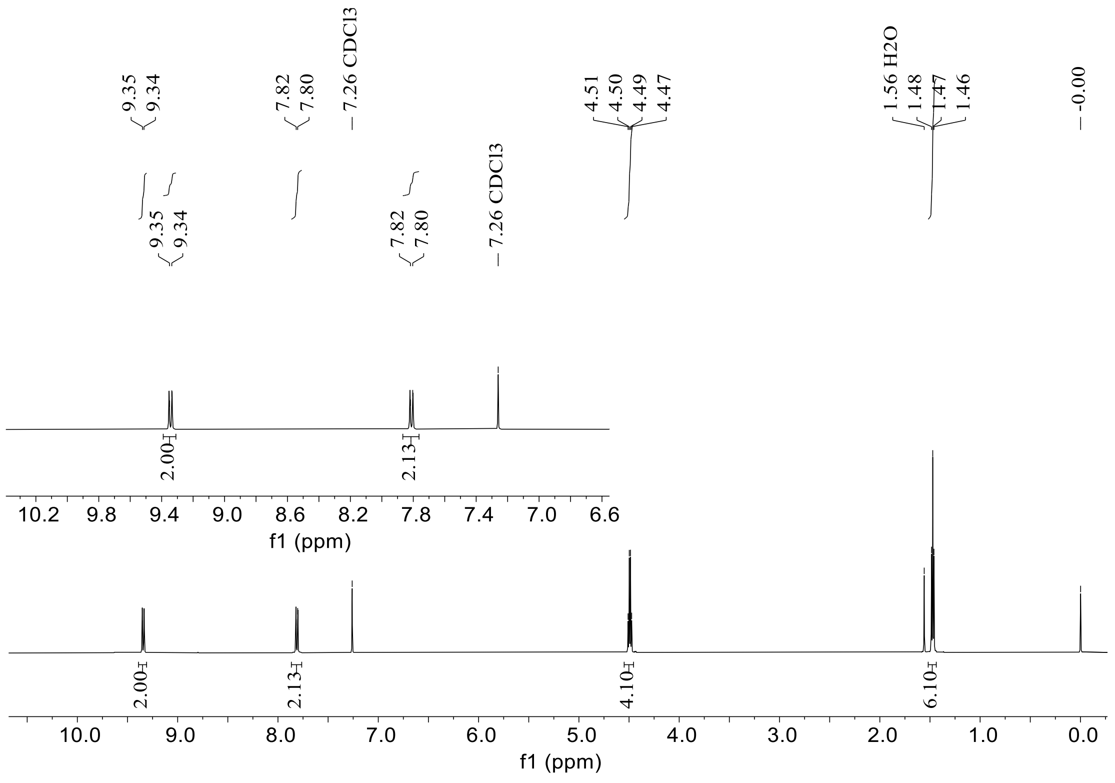

Figure S1. ^{1}H NMR spectrum of **1** (600 MHz, $CDCl_3$, 298 K).

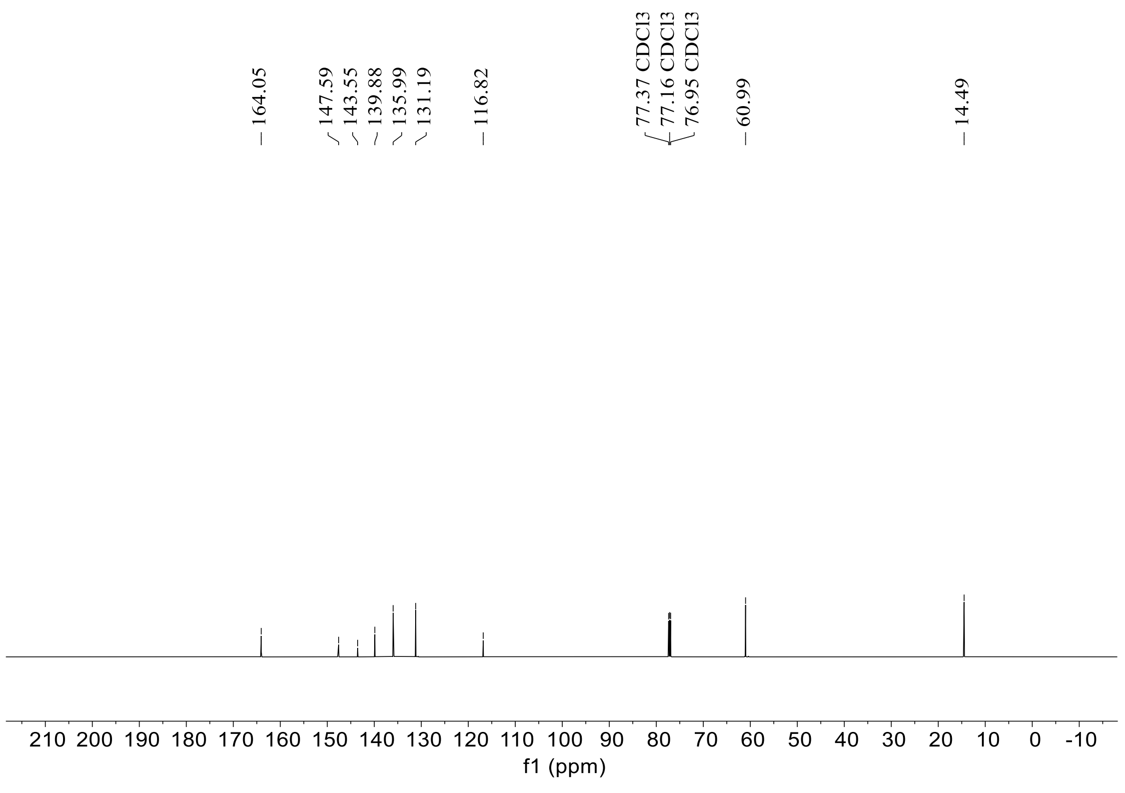

Figure S2. ^{13}C NMR spectrum of **1** (151 MHz, $CDCl_3$, 298 K).

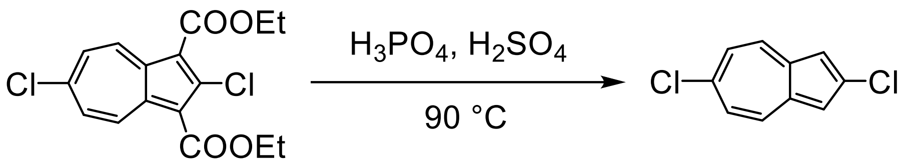

1 **2**

In a 500 mL round-bottom flask, **1** (22.50 g, 65.95 mmol) was dissolved in the mixed acidic solvent of 80 mL H_3PO_4 and 40 mL H_2SO_4. The mixture was heated at 90 °C and stirred for 30 min. The reactant was directly poured into the prepared ice water and extracted with DCM for three times. The organic layer was neutralized with diluted K_2CO_3 solution, dried with anhydrous Na_2SO_4, and the solvent was removed under vacuum. The residue was purified by silica gel column chromatography (Hexane) to give the pure compound **2** as the purple needle powder (10.00 g, 77 %).

^{1}H NMR (500 MHz, CD_2Cl_2) δ 8.05 (d, J = 10.8 Hz, 2H), 7.39 (d, J = 11.0 Hz, 2H), 7.27 (s, 2H). ^{13}C NMR (126 MHz, CD_2Cl_2) δ 144.47, 140.66, 138.44, 133.93, 125.58, 118.20.

HR-MS (EI) for **2**: calcd. for $C_{10}H_6Cl_2$ ([M]): 195.9841, found: 195.9845.

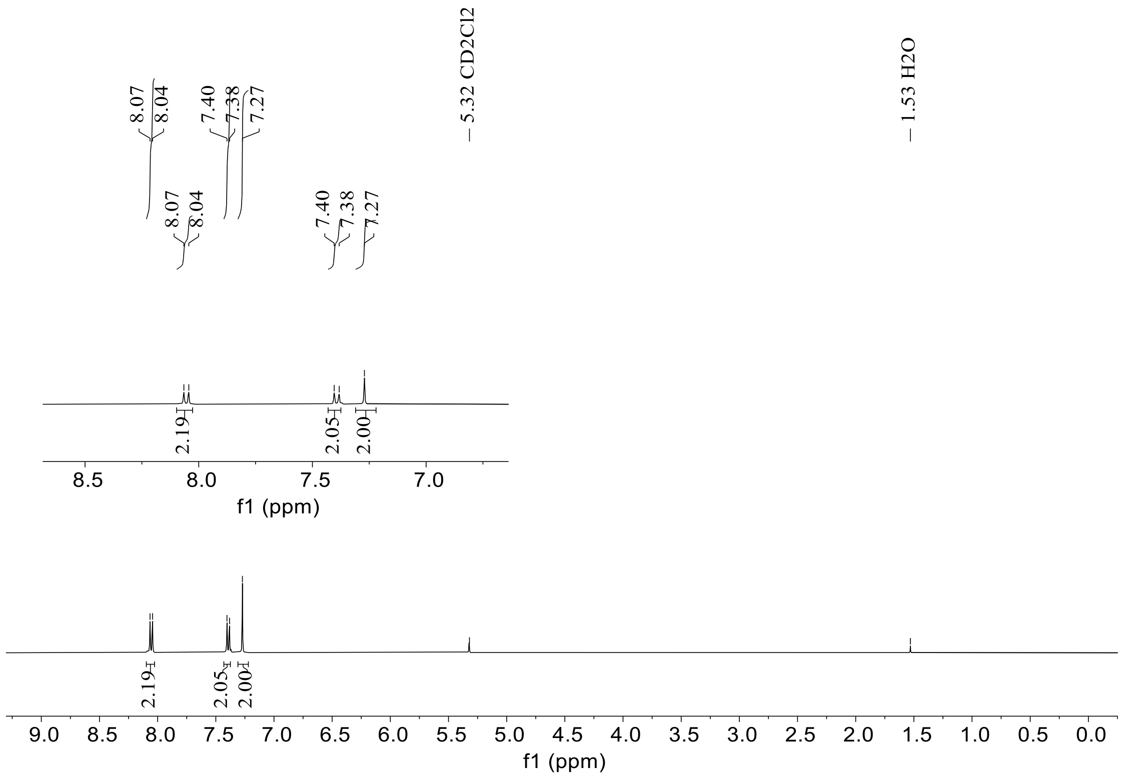

Figure S3. ^{1}H NMR spectrum of **2** (500 MHz, CD_2Cl_2, 298 K).

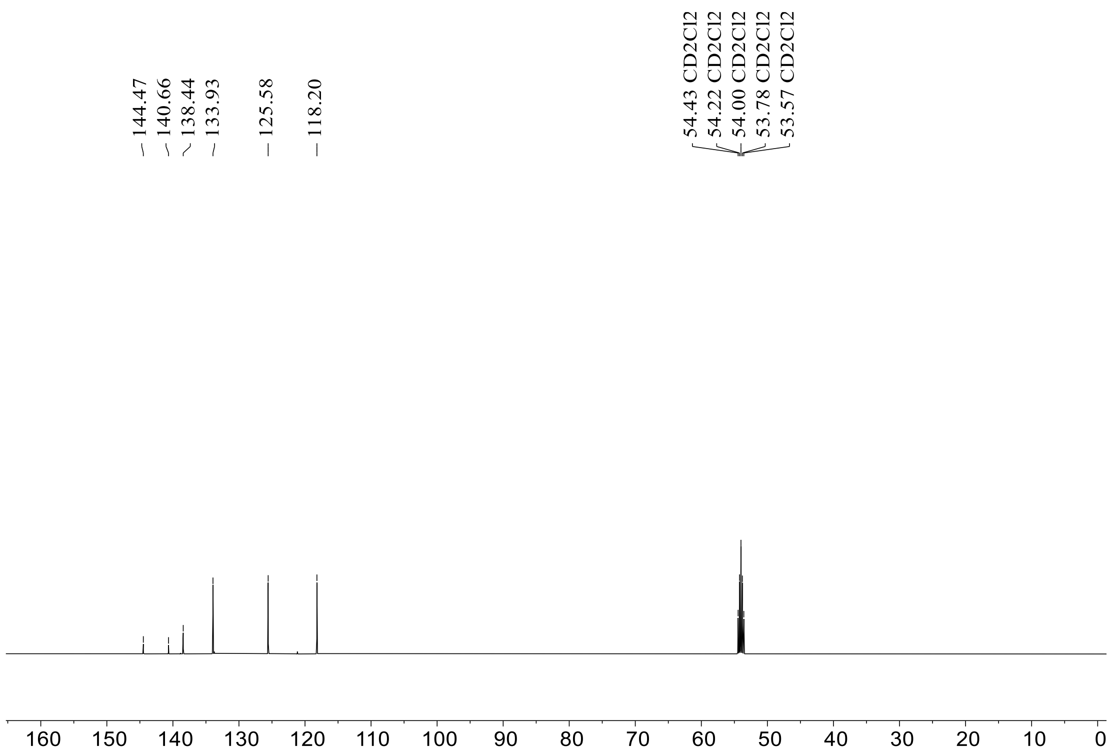

Figure S4. ^{13}C NMR spectrum of **2** (126 MHz, CD_2Cl_2, 298 K).

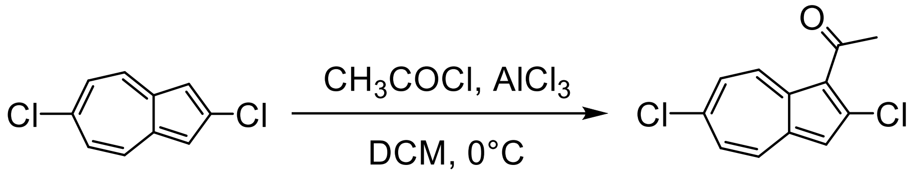

2 **3**

To a 250 mL round-bottom flask, **2** (4.00 g, 20.30 mmol) was dissolved in 200 mL DCM and then the solution was stirred at 0 °C. After adding ethanoyl chloride (1.81 mL, 25.35 mmol) and $AlCl_3$ (2.71 g, 20.32 mmol), the mixture was continuously stirred at 0 °C for 5 h. The reaction was quenched with water and filtered through the thin pad of celite (eluting with DCM). The filtrate solution was extracted with DCM with three times, dried with anhydrous Na_2SO_4, then the solvent was removed under reduced pressure. The crude product was further purified via silica gel column chromatography (Hexane/EA = 6/1) to give the pure compound **3** as the red powder (2.13 g, 44 %).

^{1}H NMR (400 MHz, $CDCl_3$) δ 9.42 (d, J = 11.0 Hz, 1H), 8.12 (d, J = 10.7 Hz, 1H), 7.69 (d, J = 11.1 Hz, 1H), 7.57 (d, J = 10.8 Hz, 1H), 7.21 (s, 1H), 2.81 (s, 3H). ^{13}C NMR (101 MHz, $CDCl_3$) δ 195.99, 146.44, 141.32, 139.89, 138.76, 136.51, 135.03, 130.23, 128.92, 123.52, 119.72, 32.39.

HR-MS (ESI) for **3**: calcd. for $C_{12}H_9Cl_2O$ ([M+H$^+$]): 239.0025, found: 239.0024.

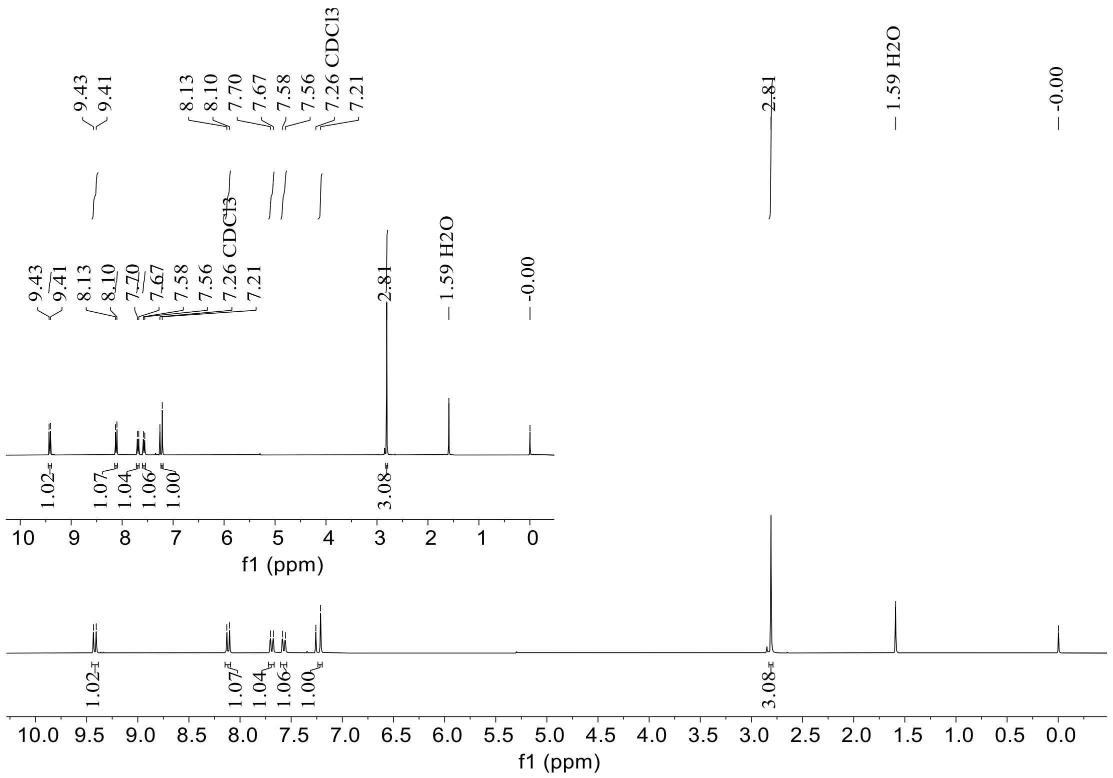

Figure S5. ^{1}H NMR spectrum of **3** (400 MHz, $CDCl_3$, 298 K).

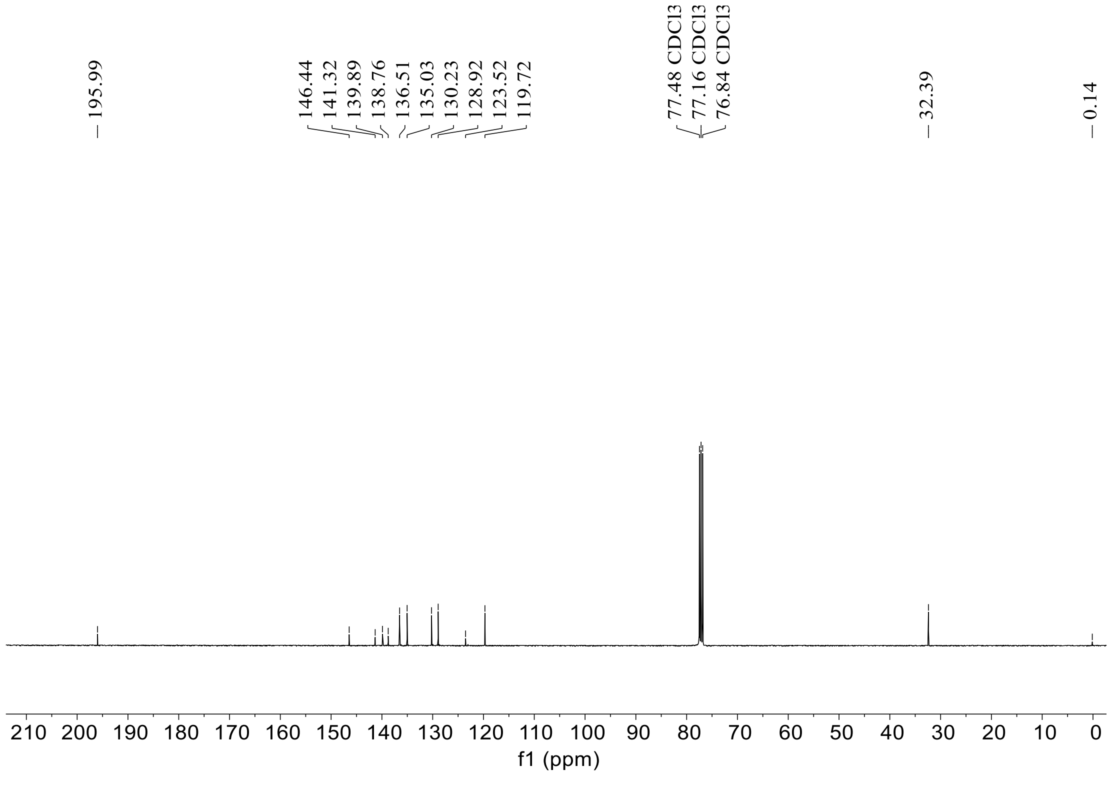

Figure S6. ^{13}C NMR spectrum of **3** (101 MHz, $CDCl_3$, 298 K).

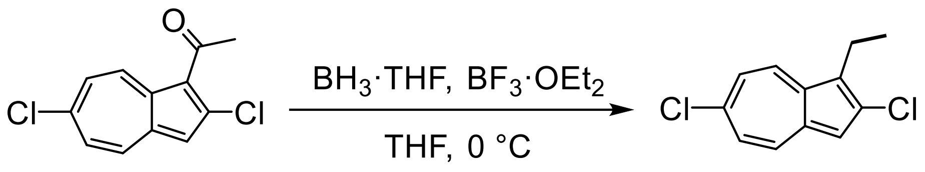

3 **4**

To a 500 mL round-bottom flask, **3** (4.27 g, 17.86 mmol) was dissolved in 150 mL THF and then the solution was stirred at 0 °C. Then the BH_3•THF (151.00 mL, 142.89 mmol) was added, followed by the BF_3•OEt_2 (31 mL, 120.55 mmol). The reaction mixture was stirred at 0 °C. After 30 min, the reactant was quenched with MeOH and then extracted with DCM and water for three times. The organic layer was then dried with anhydrous Na_2SO_4. After removing the solvent, the crude product was purified via silica gel column chromatography (Hexane) to give the pure compound **4** as the blue powder (3.30 g, 82 %).

^{1}H NMR (400 MHz, CD_2Cl_2) δ 8.04 (d, J = 10.5 Hz, 1H), 7.98 (d, J = 10.2 Hz, 1H), 7.34 – 7.26 (m, 3H), 3.04 (q, J = 7.5 Hz, 2H), 1.23 (t, J = 7.6 Hz, 3H). ^{13}C NMR (126 MHz, $CDCl_3$) δ 143.78, 139.92, 136.92, 133.42, 132.93, 130.87, 130.72, 124.01, 123.49, 116.97, 18.47, 15.42.

HR-MS for **4**: calcd. for $C_{12}H_{11}Cl_2$ ([M+H$^+$]): 225.0232, found: 225.0240.

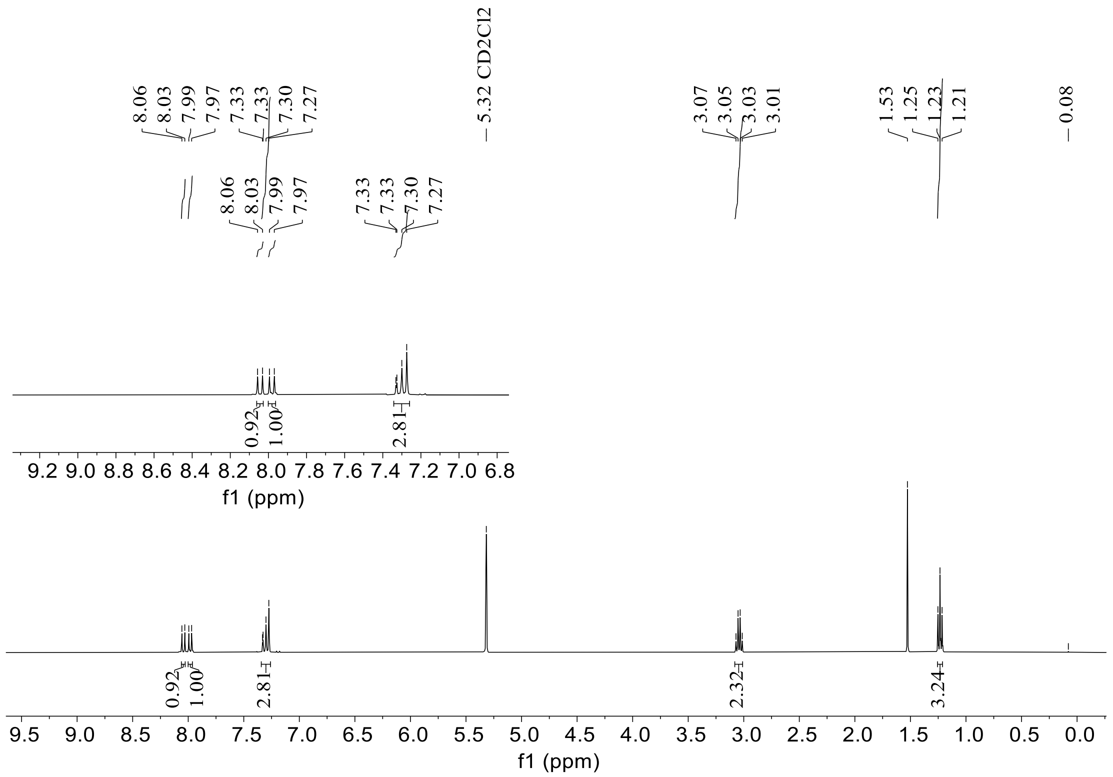

Figure S7. ^{1}H NMR spectrum of **4** (400 MHz, CD_2Cl_2, 298 K).

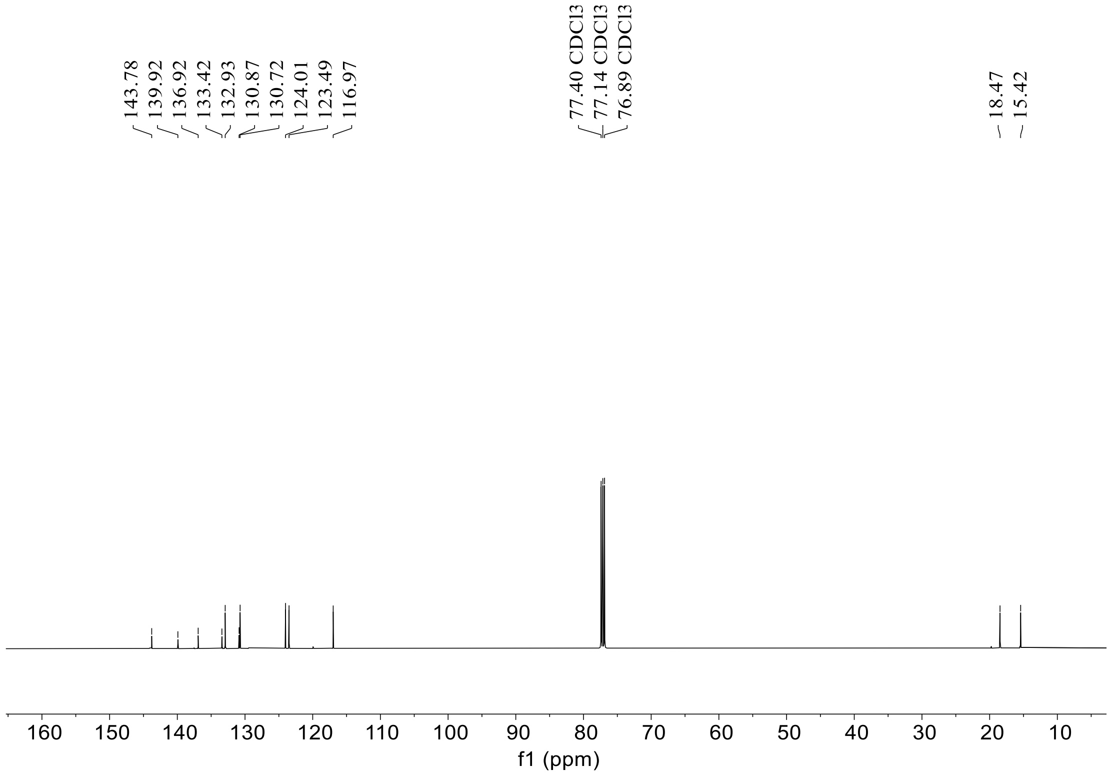

Figure S8. ^{13}C NMR spectrum of **4** (126 MHz, $CDCl_3$, 298 K).

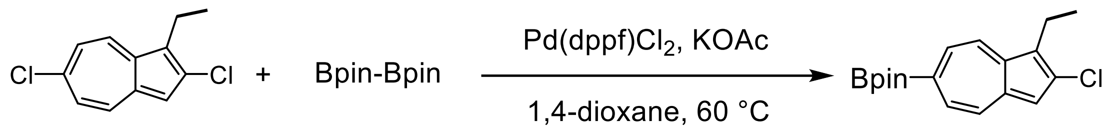

4 **5**

Compound **4** (1.50 g, 6.66 mmol), bis(pinacolato)diboron (2.54 g, 10.00 mmol) and KOAc (1.96 g, 19.97 mmol) was added into a 100 mL round-bottom flask. The flask was transferred into the glovebox, Pd(dppf)Cl_2 (487.56 mg, 0.67 mmol) and 45 mL anhydrous 1,4-dioxane were subsequently added into the flask. After sealing, the flask was removed from the glove box and then stirred at 60 °C for 12 h. Then the reactant mixture was filtered through the thin pad of celite (eluting with DCM) and the solvent was removed under reduced pressure. The obtained crude product was then purified via silica gel column chromatography (Hexane/DCM = 6/1) to give the pure compound **5** as the blue powder (1.36 g, 64 %).

^{1}H NMR (600 MHz, $CDCl_3$) δ 8.20 (d, J = 9.8 Hz, 1H), 8.15 (d, J = 9.5 Hz, 1H), 7.72 (d, J = 9.8 Hz, 1H), 7.69 (d, J = 9.6 Hz, 1H), 7.23 (s, 1H), 3.07 (q, J = 7.6 Hz, 2H), 1.39 (s, 13H), 1.25 (t, J = 7.5 Hz, 3H). ^{13}C NMR (151 MHz, $CDCl_3$) δ 141.18, 139.70, 136.01, 134.20, 131.82, 129.67, 128.92, 128.32, 114.57, 84.71, 25.05, 18.41, 15.50.

HR-MS (ESI) for **5**: calcd. for $C_{18}H_{23}BCl_2O_2$ ([M+H$^+$]): 317.1474, found: 317.1474.

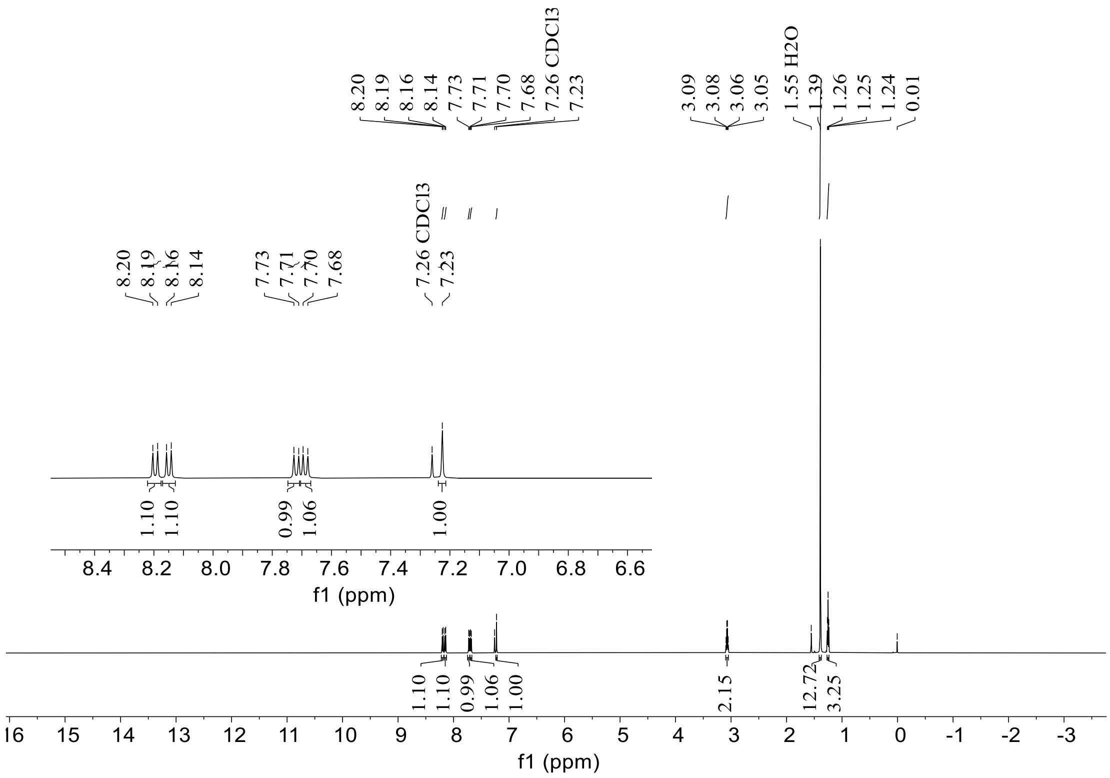

Figure S9. ^{1}H NMR spectrum of **5** (600 MHz, $CDCl_3$, 298 K).

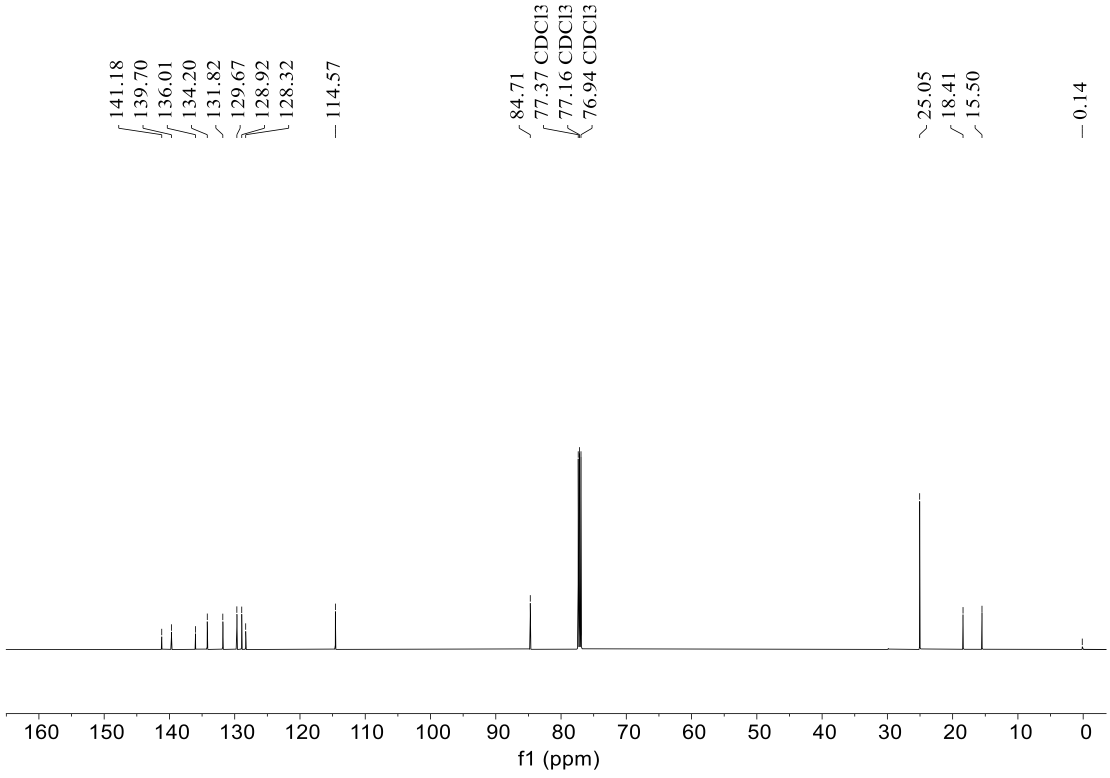

Figure S10. ^{13}C NMR spectrum of **5** (151 MHz, $CDCl_3$, 298 K).

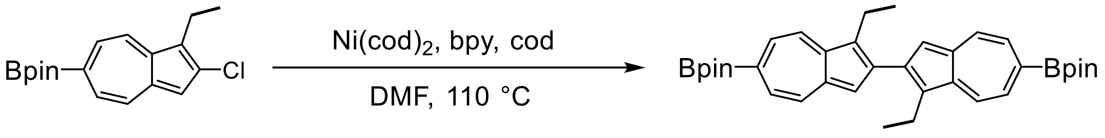

Compound **5** (500 mg, 1.58 mmol) was added into a dry 50 mL Schlenk tube. The tube was transferred into the glove box, Ni(cod)$_2$ (1.30 g, 4.73 mmol), bpy (739.92 mg, 4.73 mmol), cod (0.582 mL, 4.73 mmol) and 20 mL anhydrous DMF were subsequently added into the tube. After sealing, the tube was removed from the glovebox and then stirred at 110 °C for 24 h. Then the reaction mixture was filtered through the thin pad of celite (eluting with EA) and removed the solvent. The crude product was then purified via gel permeation chromatography (eluent: THF) to obtain the pure target **6** as the green powder (232.00 mg, 52 %).

^{1}H NMR (600 MHz, $CDCl_3$) δ 8.33 (d, J = 9.7 Hz, 2H), 8.26 (d, J = 9.6 Hz, 2H), 7.68 (d, J = 9.7 Hz, 2H), 7.65 (d, J = 9.5 Hz, 2H), 7.49 (s, 2H), 3.19 (q, J = 7.5 Hz, 4H), 1.41 (s, 24H), 1.20 (t, J = 7.5 Hz, 6H). ^{13}C NMR (151 MHz, $CDCl_3$) δ 148.19, 141.09, 137.73, 134.94, 132.55, 131.47, 128.66, 127.85, 117.80, 84.58, 25.07, 19.14, 16.83.

HR-MS (ESI) for 3: calcd. for $C_{36}H_{45}B_2O_4$ ([M+H$^+$]): 563.3498, found: 563.3501.

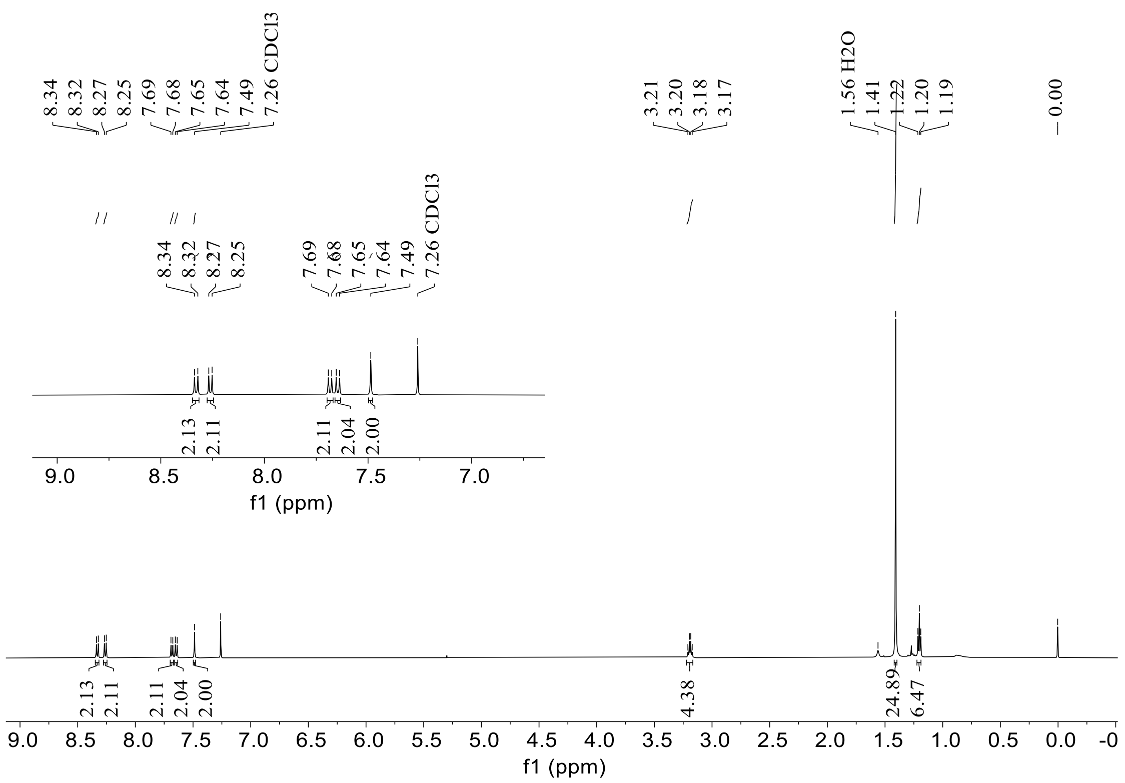

Figure S11. ^{1}H NMR spectrum of **6** (600 MHz, $CDCl_3$, 298 K).

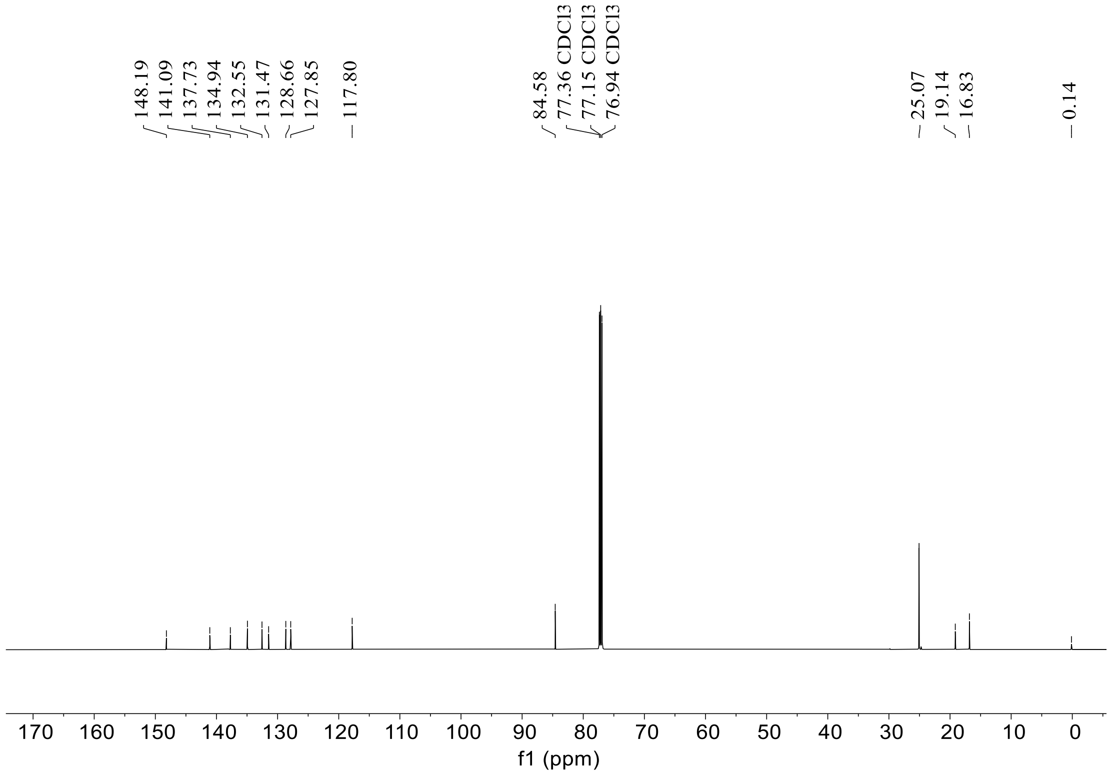

Figure S12. ^{13}C NMR spectrum of **6** (151 MHz, $CDCl_3$, 298 K).

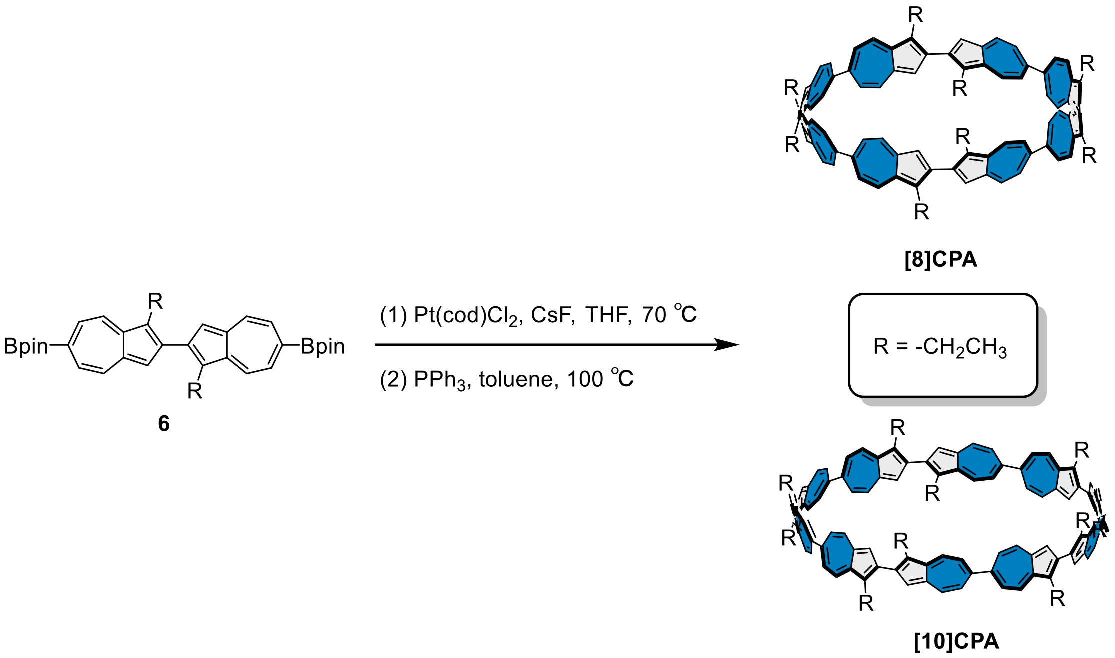

Compound **6** (100.00 mg, 0.18 mmol) was added into a dry 35 mL Schlenk pressure tube. The tube was transferred into the glovebox, and then Pt(cod)Cl_2 (67.00 mg, 0.18 mmol), CsF (270.00 mg, 1.80 mmol) and 18 mL anhydrous THF were added subsequently. After sealing, the tube was removed from the glove box and then stirred at 70 °C for 24 h. Then the solvent was removed under vacuum and the residue was transferred into the glove box again. After adding PPh_3 and 18 mL anhydrous toluene, the mixture was stirred at 100 °C for 24 h. The reactant was then poured into MeOH and filtered to collect the solid. The crude product was purified by silica gel column chromatography (Hexane/DCM = 3/1) to afford the pure **[8]CPA** (1.00 mg, 2 %) and **[10]CPA** (< 1mg, < 1 %) simultaneously.

[8]CPA: ^{1}H NMR (400 MHz, CD_2Cl_2+CS_2) δ 8.32 (d, J = 10.7 Hz, 8H), 7.91 (d, J = 10.1 Hz, 8H), 7.53 (d, J = 10.5 Hz, 8H), 7.11 (s, 8H), 6.95 (d, J = 10.1 Hz, 8H), 3.46 (q, J = 7.5 Hz, 16H), 1.46 (d, J = 14.9 Hz, 24H). ^{13}C NMR (151 MHz, CD_2Cl_2+CS_2) δ 193.06, 153.41, 146.57, 140.66, 136.40, 134.30, 133.82, 132.56, 127.76, 121.97, 120.26, 20.16, 17.08.

HR-MS (MALDI-TOF) for **[8]CPA**: calcd. for $C_{96}H_{80}$([M]): 1232.6260, found: 1232.6281.

[10]CPA: ^{1}H NMR (400 MHz, CD_2Cl_2) δ 8.38 (d, J = 10.6 Hz, 10H), 8.05 (d, J = 10.3

Hz, 10H), 7.53 (d, J = 10.7 Hz, 10H), 7.26 (s, 10H), 7.09 (d, J = 10.1 Hz, 10H), 3.46 – 3.38 (m, 20H), 1.40 (t, J = 7.4 Hz, 30H). ^{13}C NMR (151 MHz, CD_2Cl_2+CS_2) δ 153.96, 146.82, 140.09, 136.47, 134.60, 133.70, 132.59, 126.71, 122.66, 119.93, 19.89, 17.05. HR-MS (MALDI-TOF) for **[10]CPA**: calcd. for $C_{96}H_{80}$([M]): 1540.7825, found: 1540.7744.

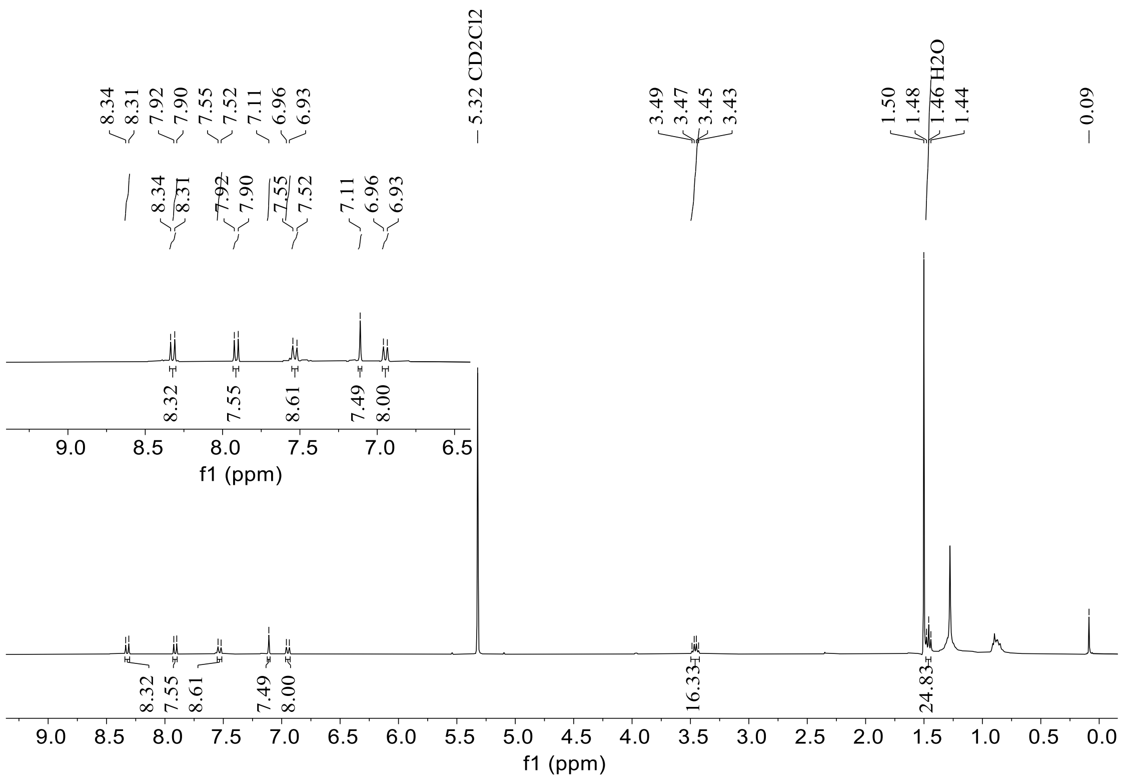

Figure S13. ^{1}H NMR spectrum of **[8]CPA** (400 MHz, CD_2Cl_2+CS_2, 298 K).

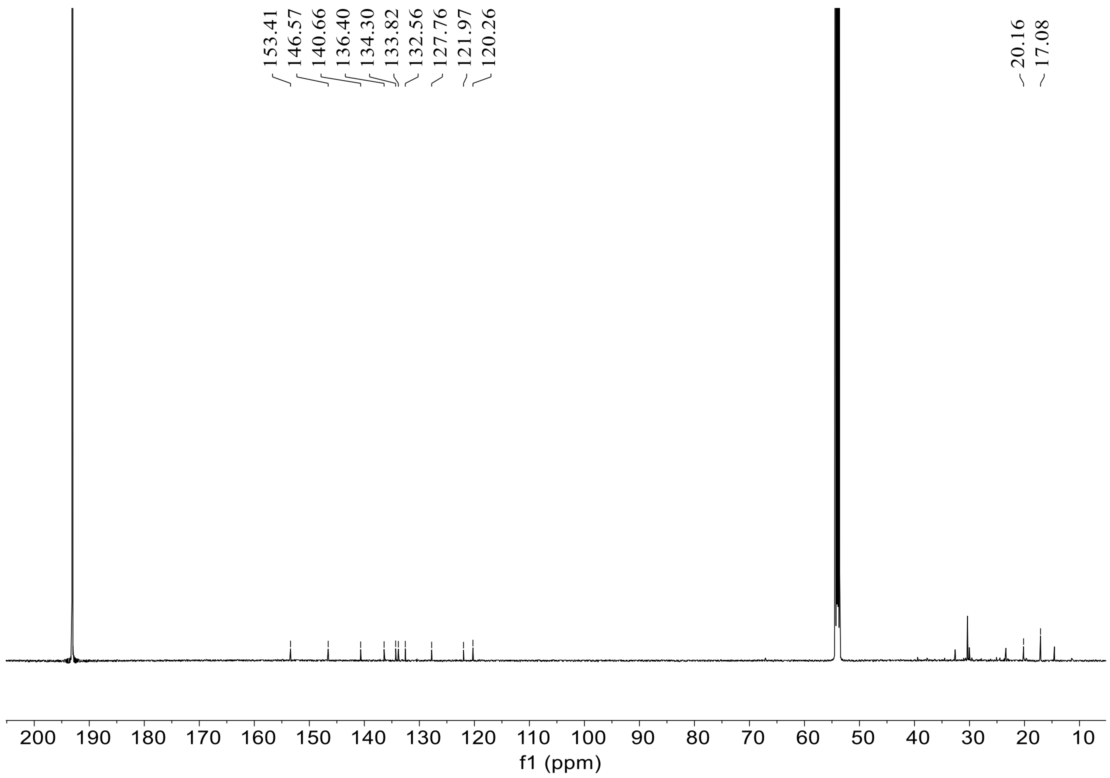

Figure S14. ^{13}C NMR spectrum of **[8]CPA** (151 MHz, CD_2Cl_2+CS_2, 298 K).

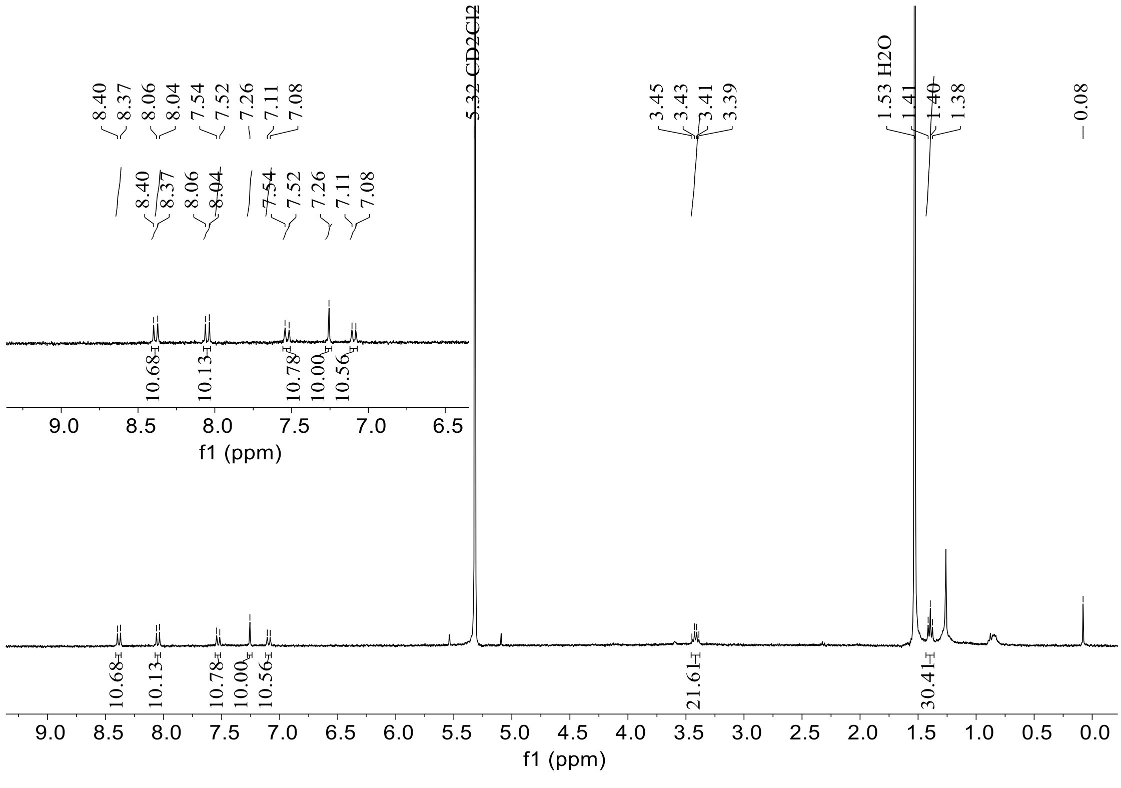

Figure S15. ^{1}H NMR spectrum of **[10]CPA** (400 MHz, CD_2Cl_2, 298 K).

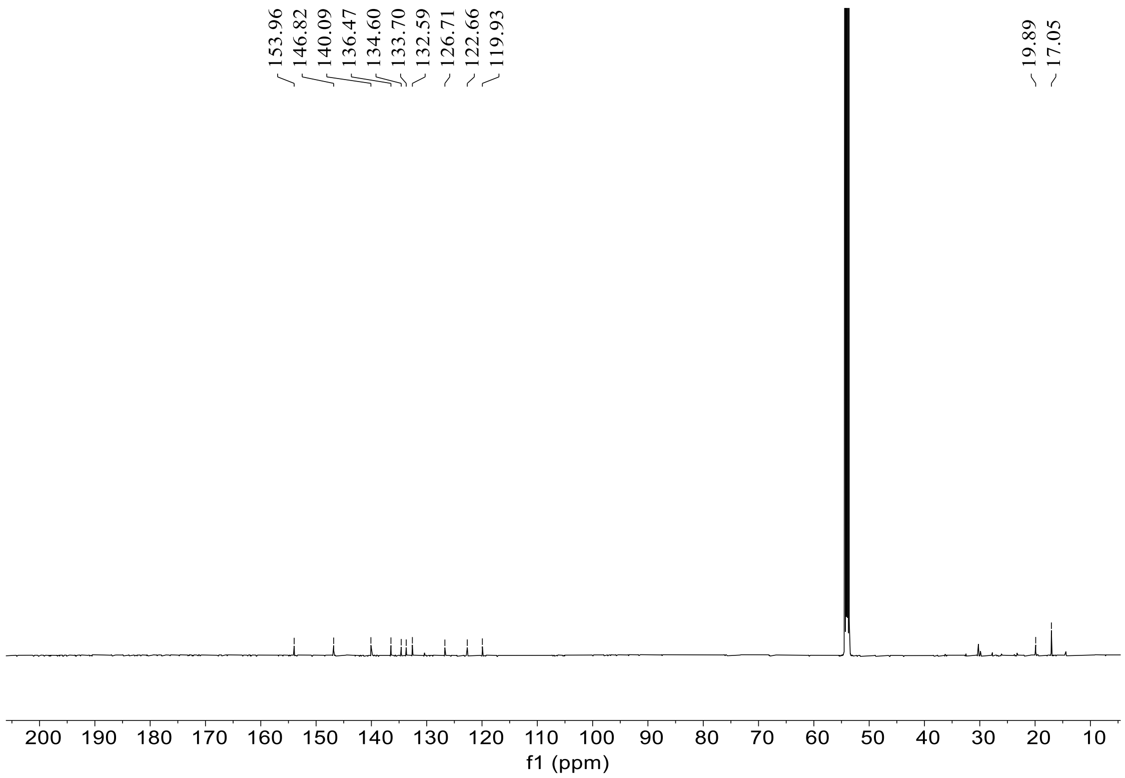

Figure S16. ^{13}C NMR spectrum of **[10]CPA** (151 MHz, CD_2Cl_2, 298 K).

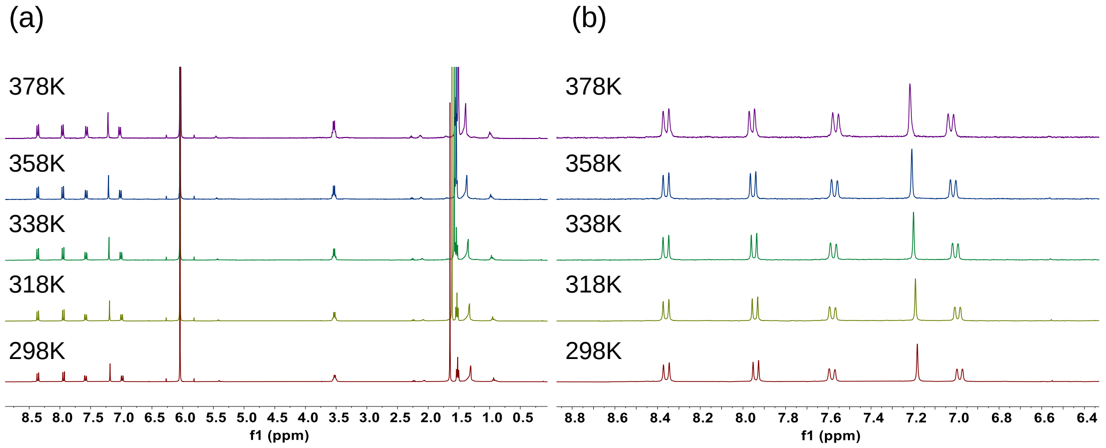

Figure S17. (a) and (b) The complete and enlarged aromatic region of high-temperature ^{1}H NMR spectra of **[8]CPA**.

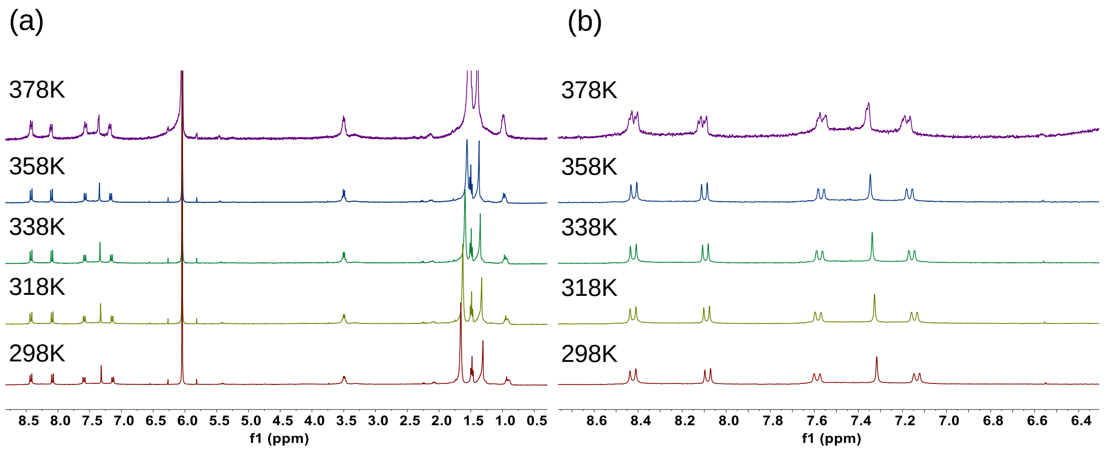

Figure S18. (a) and (b) The complete and enlarged aromatic region of high-temperature ^{1}H NMR spectra of **[10]CPA**.

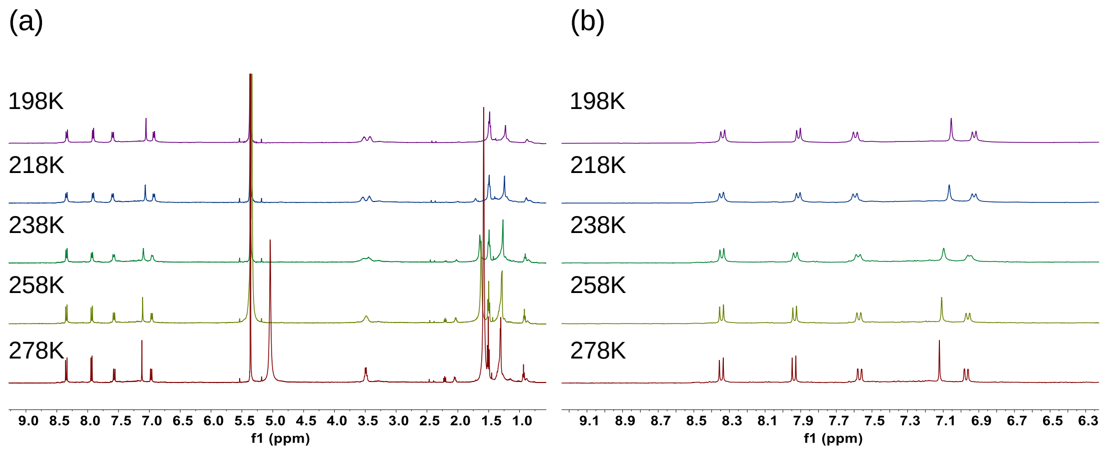

Figure S19. (a) and (b) The complete and enlarged aromatic region of low-temperature ^{1}H NMR spectra of **[8]CPA**.

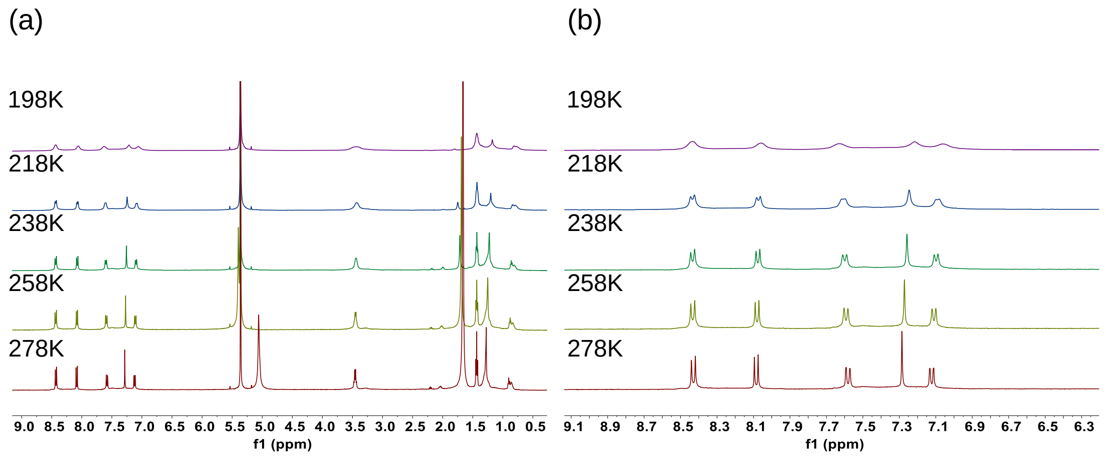

Figure S20. (a) and (b) The complete and enlarged aromatic region of low-temperature ^{1}H NMR spectra of **[10]CPA**.

As shown in Figure S17 and 18, the NMR signals exhibit slightly changes as the

temperature increases, while only the signals of **[10]CPA** at 373K showing broad peaks owing to the faster rotation. Upon lowering the temperature from 278K to 198K (Figure S19 and S20), the signals of both CPAs shift slightly up-field, accompanied by lower peak intensity and broader peaks. These observations result from decreased solubility and slower rotation of CPAs. Additionally, the signal at around 5 ppm under 278K originates from the interactions of water molecules[2].

3. X-ray crystallography

The X-ray crystallographic coordinates for these two crystalline structures reported in this work have been collected at Cambridge Crystallographic Data Centre (CCDC), under collecting number, CCDC 2530366 (for **2**) and CCDC 2552260 (for **[8]CPA**). Both data of those two crystalline structures can be achieved free of charge from CCDC via https://www.ccdc.cam.ac.uk/structures/.

Single-crystal X-ray diffraction analysis of **2** was performed at 100 K using Cu Kα radiation (λ = 1.54178 Å), revealing that the compound crystallizes in the monoclinic system with space group P121/n1. The crystal size was approximately 0.1 x 0.07 x 0.03 mm^3.The unit cell parameters are a = 9.5922 Å, b = 3.8323 Å, c = 11.4434 Å, V = 420.08 $Å^3$, with Z = 2. The refinement converged with R1 = 0.0266 (for $I \geq 2\sigma(I)$) and wR2 = 0.0815 for all data, indicating a reliable structural solution. The calculated density is 1.558 g/cm^3.

Single-crystal X-ray diffraction analysis of **[8]CPA** was performed at 300 K using Ga Kα radiation (λ = 1.34139 Å), revealing that the compound crystallizes in the tetragonal system with space group P4/nnc. The crystal size was approximately 0.16 × 0.13 × 0.12 mm^3.The unit cell parameters are a = 28.310 Å, b = 28.310 Å, c = 6.3427 Å, V = 5083 $Å^3$, with Z = 2. The refinement converged with R1 = 0.0905 (for $I \geq 2\sigma(I)$) and wR2 = 0.2453 for all data, indicating a reliable structural solution. The calculated density is 0.806 g/cm^3.

Table S1. Crystal data and structure refinement for **2**.

Identification code	**2**	
Empirical formula	C10H6Cl2	
Formula weight	197.05	
Temperature	100 K	
Wavelength	1.54178 Å	
Crystal system	monoclinic	
Space group	P121/n1	
Unit cell dimensions	a = 9.5922 (6) Å	α= 90 °.
	b = 3.8323 (2) Å	β= 93.002 (3)°.
	c = 11.4434 (7) Å	γ = 90 °.
Volume	420.08 (4) $Å^3$	
Z	2	
Density (calculated)	1.558 g/cm^3	
Absorption coefficient	6.372 mm^{-1}	
F(000)	200.0	
Crystal size	0.1 x 0.07 x 0.03 mm^3	
Theta range for data collection	5.870 to 68.414 °.	
Index ranges	-11 ≤ h ≤ 11, -4 ≤ k ≤ 4, -13 ≤ l ≤ 13	
Reflections collected	5959	
Independent reflections	764 [R(int) = 0.1424]	
Completeness to theta = 68.414°	99.7 %	
Data / restraints / parameters	775 / 39 / 96	
Goodness-of-fit on F^2	1.173	
Final R indices [I>2sigma(I)]	R1 = 0.0266, wR2 = 0.0815	
R indices (all data)	R1 = 0.0268, wR2 = 0.0817	

Table S2. Crystal data and structure refinement for **[8]CPA.**

Identification code	**[8]CPA**
Empirical formula	C96H80
Formula weight	1233.60
Temperature/K	300
Crystal system	tetragonal
Space group	P4/nnc
a/Å	28.310(5)
b/Å	28.310(5)
c/Å	6.3427(17)
α/°	90
β/°	90
γ/°	90
Volume/Å3	5083(2)
Z	2
ρcalcg/cm3	0.806
μ/mm-1	0.218
F(000)	1312
Crystal size/mm3	0.16 × 0.13 × 0.12
Radiation	GaKα (λ = 1.34139)
2Θ range for data collection/°	5.48 to 63.96
Index ranges	-32 ≤ h ≤ 32, -32 ≤ k ≤ 32, -7 ≤ l ≤ 7
Reflections collected	49350
Data/restraints/parameters	1966/81/110
Final R indexes [I>=2σ (I)]	R1 = 0.0905, wR2 = 0.2143
Final R indexes [all data]	R1 = 0.1515, wR2 = 0.2453

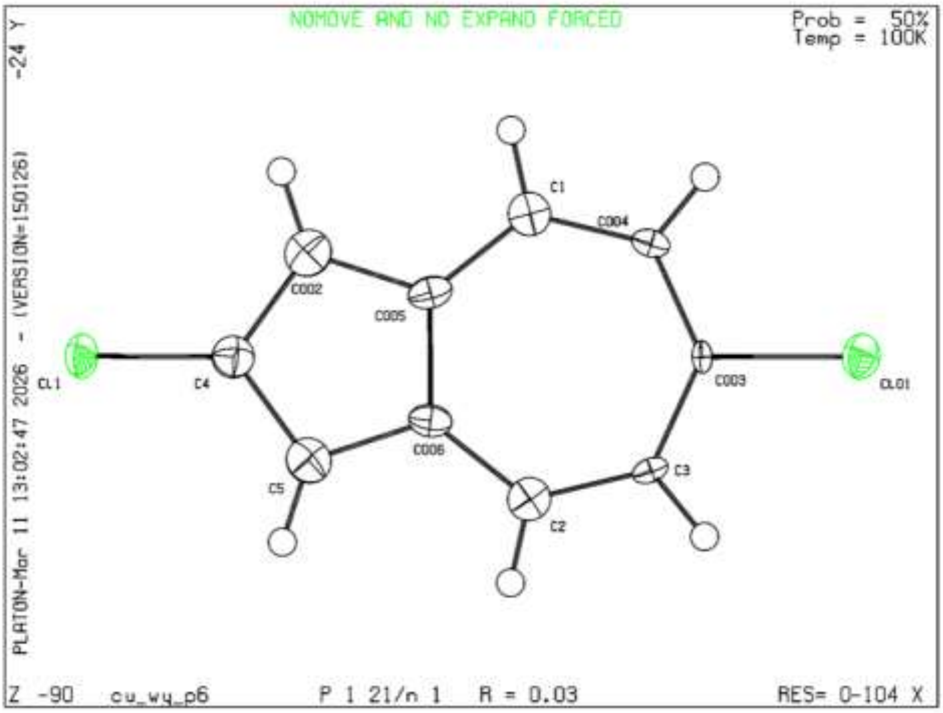

Figure S21. Thermal ellipsoid plot of **2** with ellipsoids drawn at the 50 % probability level.

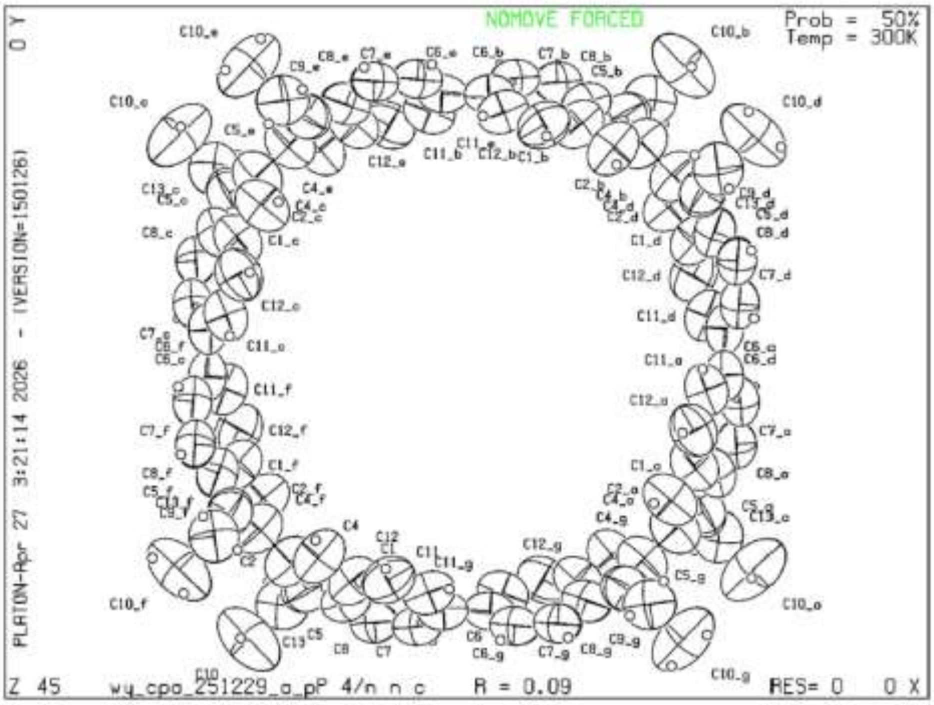

Figure S22. Thermal ellipsoid plot of **[8]CPA** with ellipsoids drawn at the 50 % probability level.

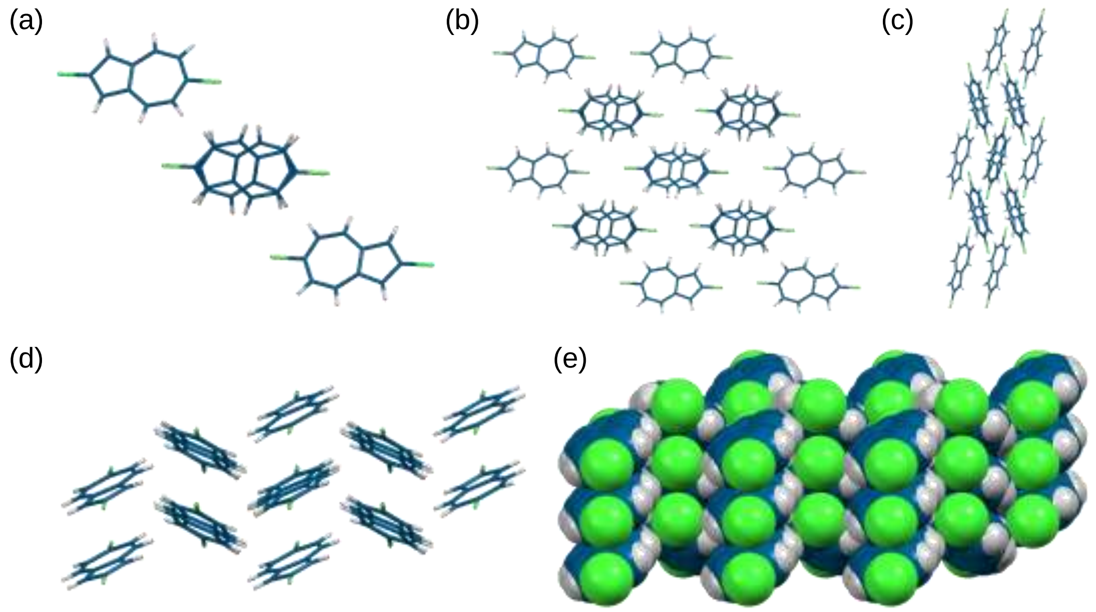

Figure S23. (a) Repeating unit of **2** in one unit cell from. (b), (c) and (d) View of 2x2x2 packing of **2** down b, a and c axis. (3) View of 3x3x3 packing of **2** down c axis.

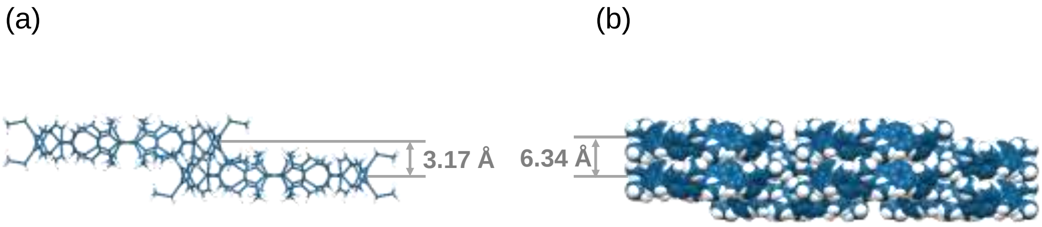

Figure S24. (a) Repeating unit of **[8]CPA** in one unit cell from the side view. (b) Side view of the packing structure of **[8]CPA**.

4. Theoretical Analysis

All density functional theory (DFT) calculation was carried out by using the Gaussian 16 program[3]. The B3LYP functional was used for geometry optimization in the ground state, and the 6-31G(d) basis set was used. The NCI plot was calculated at B3LYP/6-31G(d) level. The strain energies were also calculated and analyzed via Strainviz based on B3LYP/6-31G(d) level[4]. Nucleus independent chemical shifts (NICS) values were calculated using the standard gauge invariant atomic orbital (GIAO)[5-7] method at B3LYP functional and all NICS values were averaged by two positions (above and below the plane) of each monocycles with help of py Aroma-4[8]. Uv-vis absorption spectra were calculated at PBE1PBE/6-311G(d) basis set using DCM as solvent. Calculated maps were visualized with the help of Multiwfn[9] and VMD[10].

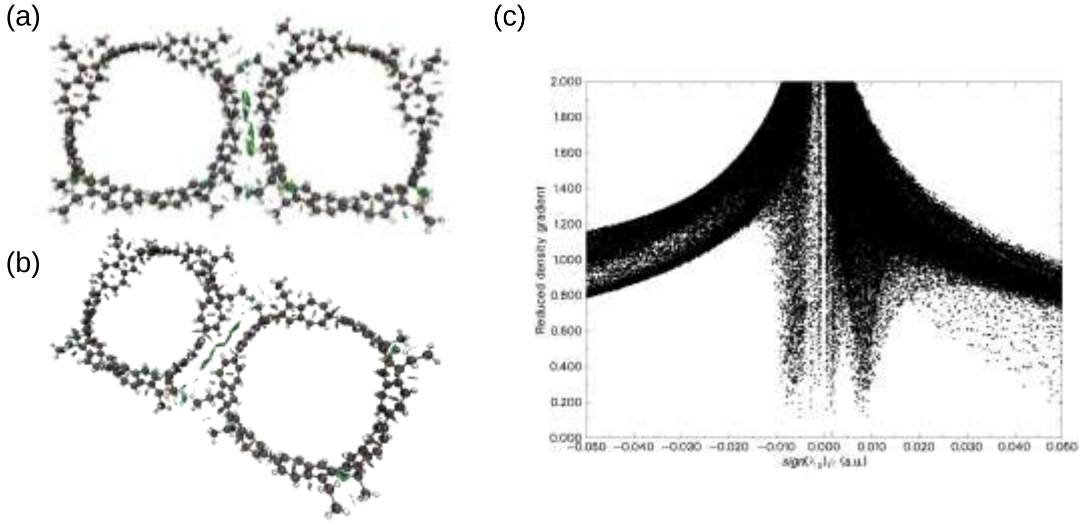

Figure S25. (a) and (b) Top and side view of NCI plot of **[8]CPA**, respectively. Green isosurfaces presents intermolecular weak van der Waals interactions. (c) Plots of the reduced density gradient versus the electron density multiplied by the sign of the second Hessian eigenvalue for **[8]CPA**. The data was obtained by evaluating B3LYP/6-31G(d) density or promolecular density and gradient values on cuboid grids.

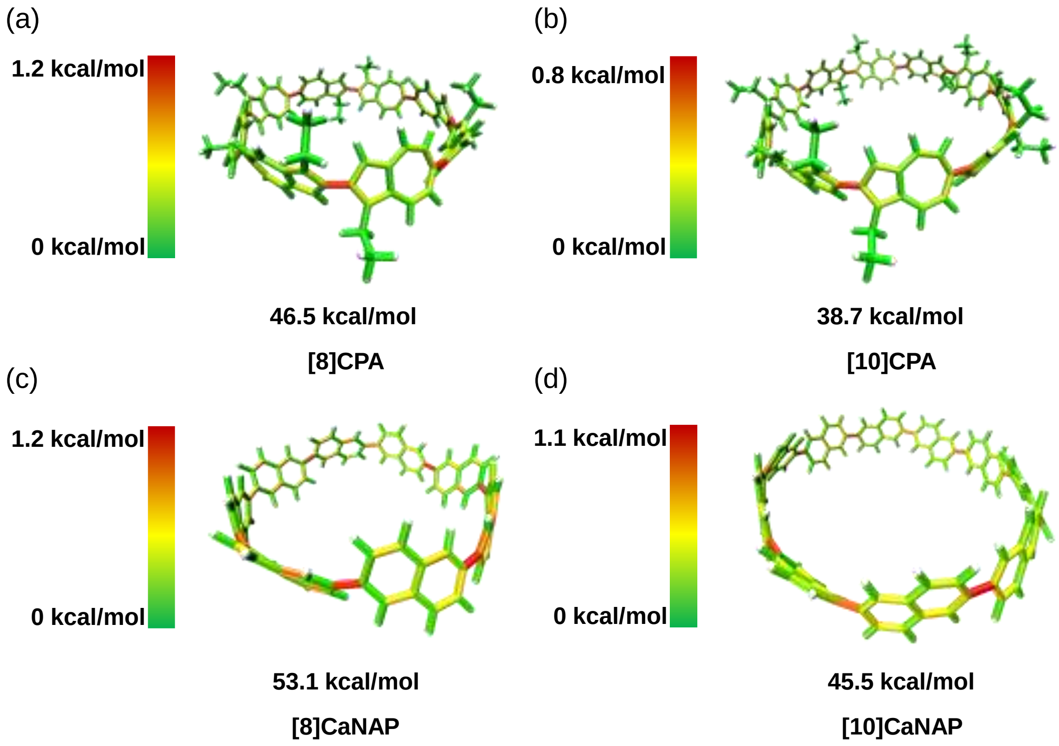

Figure S26. (a), (b), (c) and (d) Strain energies of **[8]CPA**, **[10]CPA**, [8]CaNAP and [10]CaNAP calculated at B3LYP/6-31G(d) level via Strainviz, respectively.

Table S3. The calculated total, bond, angle and dihedral strain of **[8]CPA**, **[10]CPA**, [8]CaNAP and [10]CaNAP.

Strain Energies (kcal/mol)	Total Strain	Bond Strain	Angle Strain	Dihedral Strain
[8]CPA	46.5	2.6	4.5	39.5
[10]CPA	38.7	1.5	2.7	34.5
[8]CaNAP	53.1	13.9	1.2	37.9
[10]CaNAP	45.5	6.1	2.1	37.2

Table S4. The calculated dipole moment of **[8]CPA** and **[10]CPA**, respectively.

Dipole moment (Debye)	X	Y	Z	Total
[8]CPA	0.0978	0.0337	0.0140	0.1043
[10]CPA	0.0520	0.1022	0.0273	0.1178

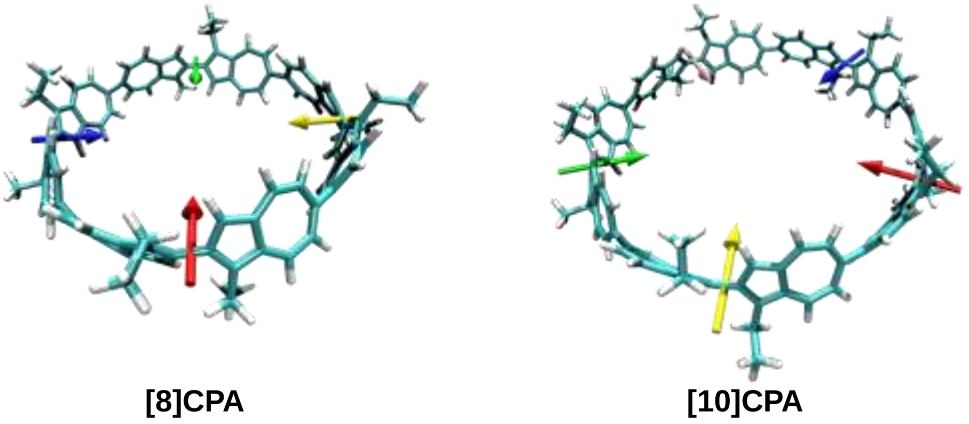

Figure S27. Visualization of calculated dipole moment of the segments in **[8]CPA** and **[10]CPA**, respectively.

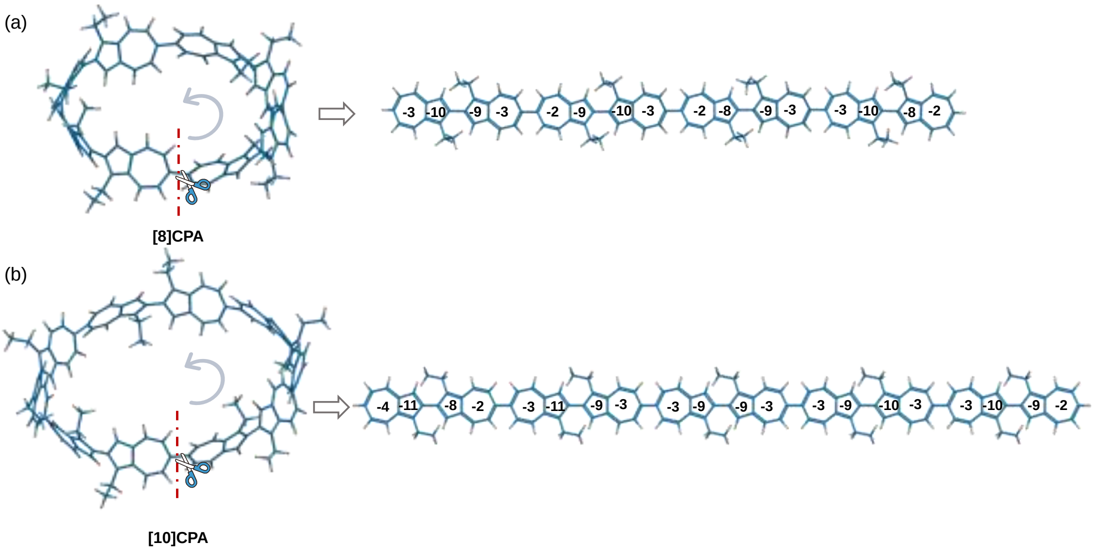

Figure S28. (a-b) Calculated NICS $(1)_{zz\text{-}avg}$ values of **[8]CPA** and **[10]CPA**.

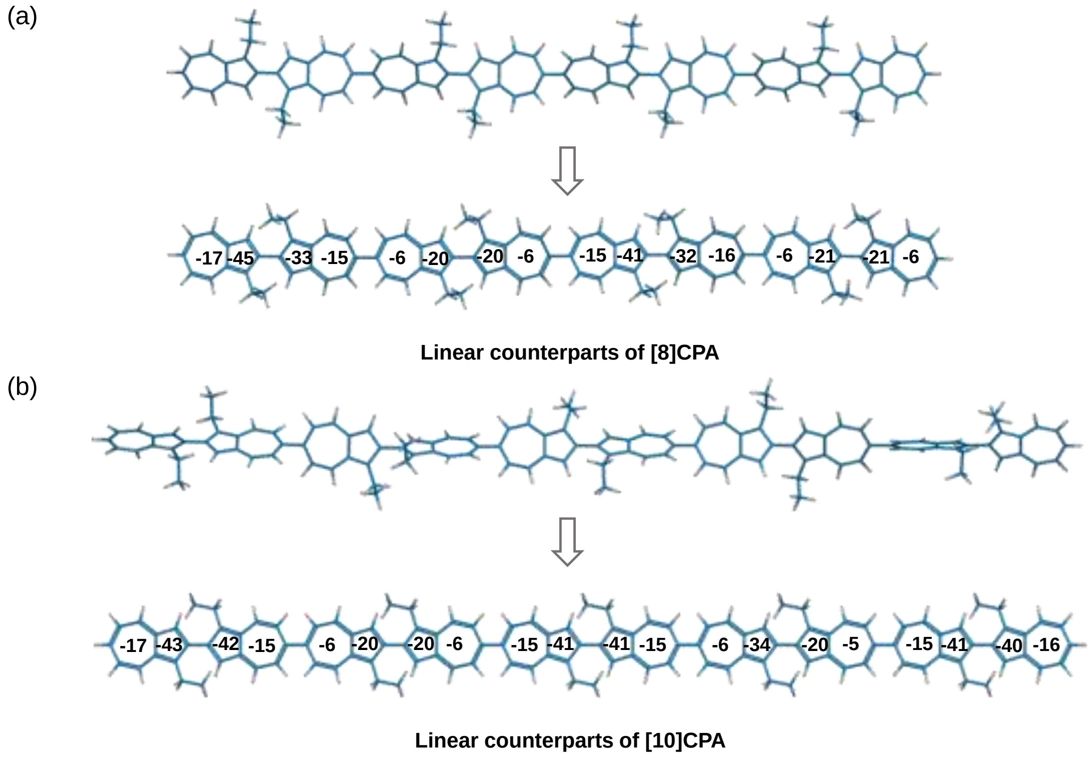

Figure S29. (a-b) Calculated NICS $(1)_{zz\text{-avg}}$ values of linear counterparts of **[8]CPA** and **[10]CPA.**

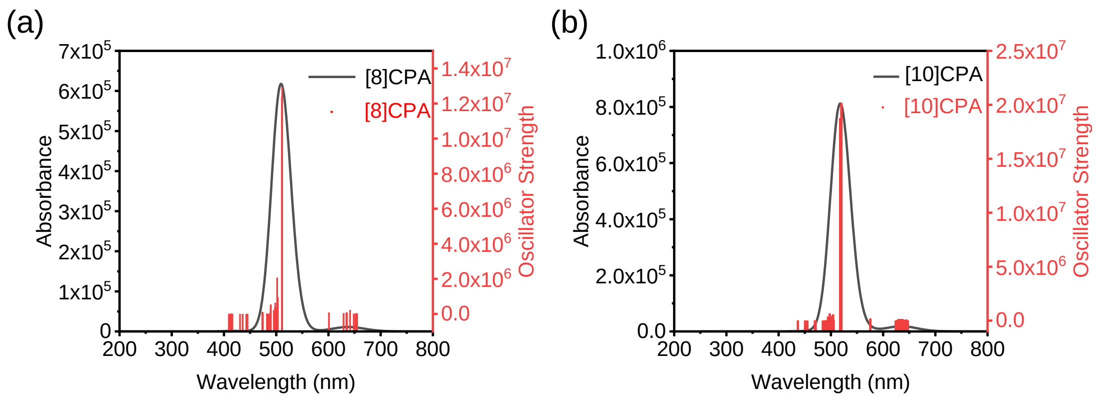

Figure S30. (a-b) Simulated UV-vis absorption spectra of **[8]CPA** and **[10]CPA** at PBE1PBE/6-311G(d) using DCM as solvent.

5. CV, UV-vis absorption and fluorescence spectra

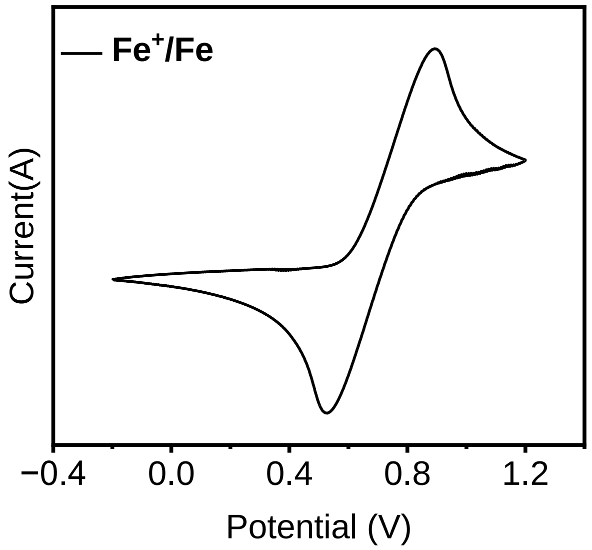

Figure S31. CV spectrum of Ferrocene in DCM.

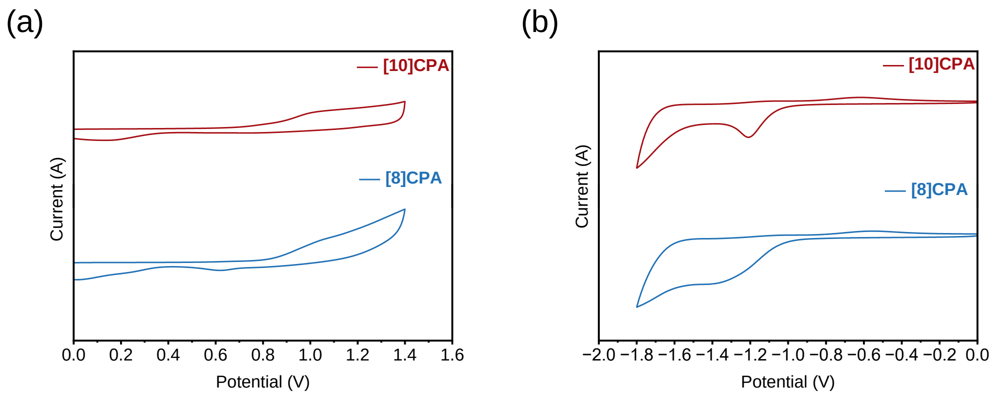

Figure S32. (a) CV spectra of oxidation region of **[8]CPA** and **[10]CPA** measured in DCM. (b) CV spectra of reduction region of **[8]CPA** and **[10]CPA** measured in DCM. Cyclic voltammetry (CV) measurements of both CPAs were performed in DCM (Figure S31, 32). **[8]CPA** and **[10]CPA** display obvious reduction potentials at -1.02 and -1.05 V, respectively.

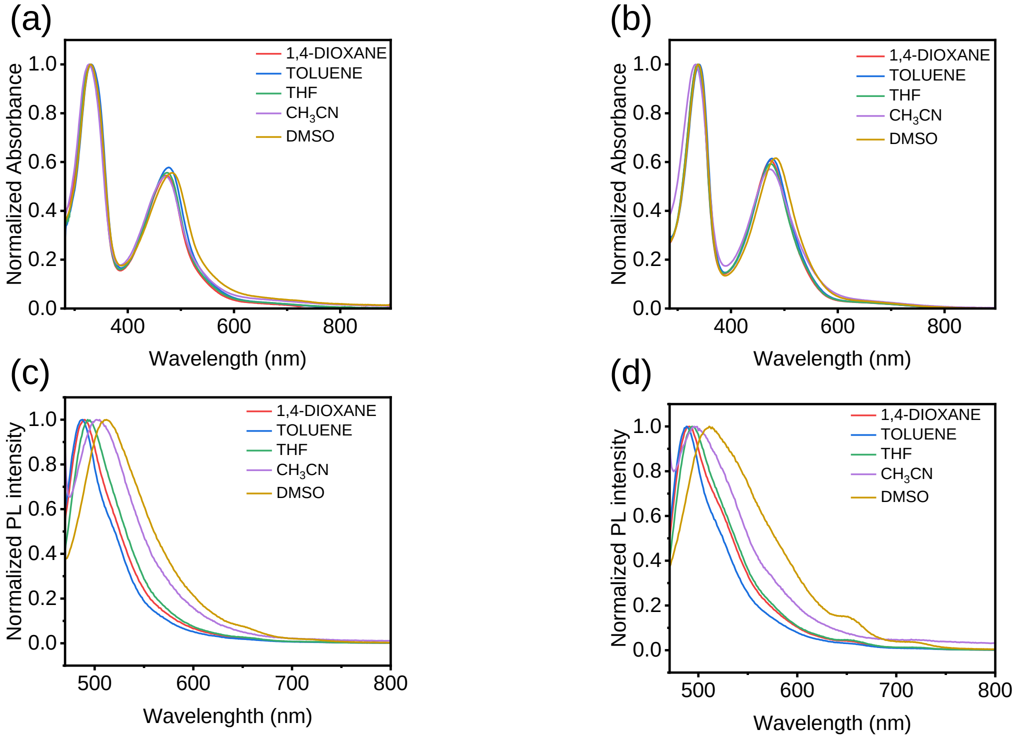

Figure S33. (a) and (b) Normalized UV-vis absorption spectra of **[8]CPA** and **[10]CPA** in varying polarity solvents, respectively. (c) and (d) Normalized fluorescence spectra of **[8]CPA** and **[10]CPA** in varying polarity solvents, respectively.

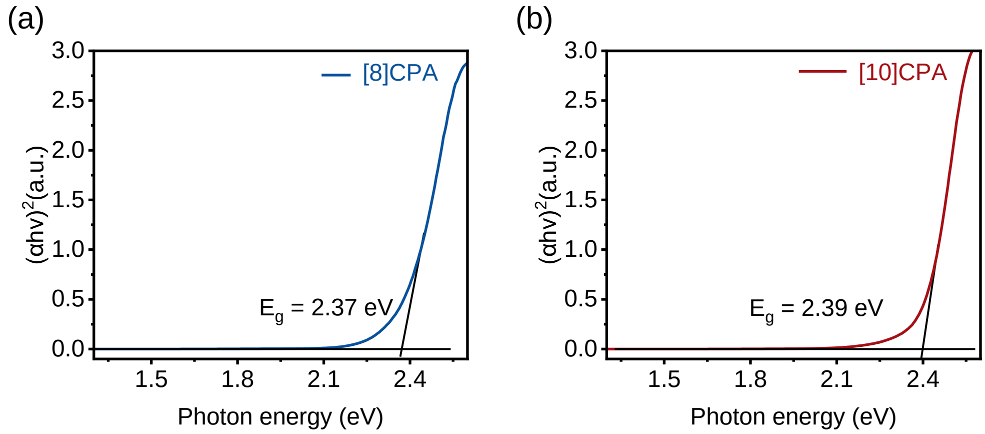

Figure S34. (a) and (b) Tauc plot of **[8]CPA** and **[10]CPA**, respectively.

6. High-resolution mass spectrometry

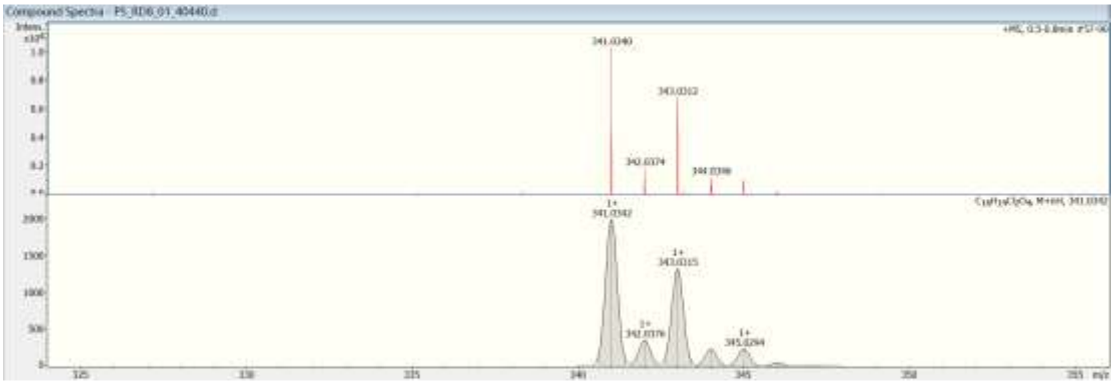

Figure S35. HR-MS (ESI) spectra for **1** (Red: experiment; Black: simulation).

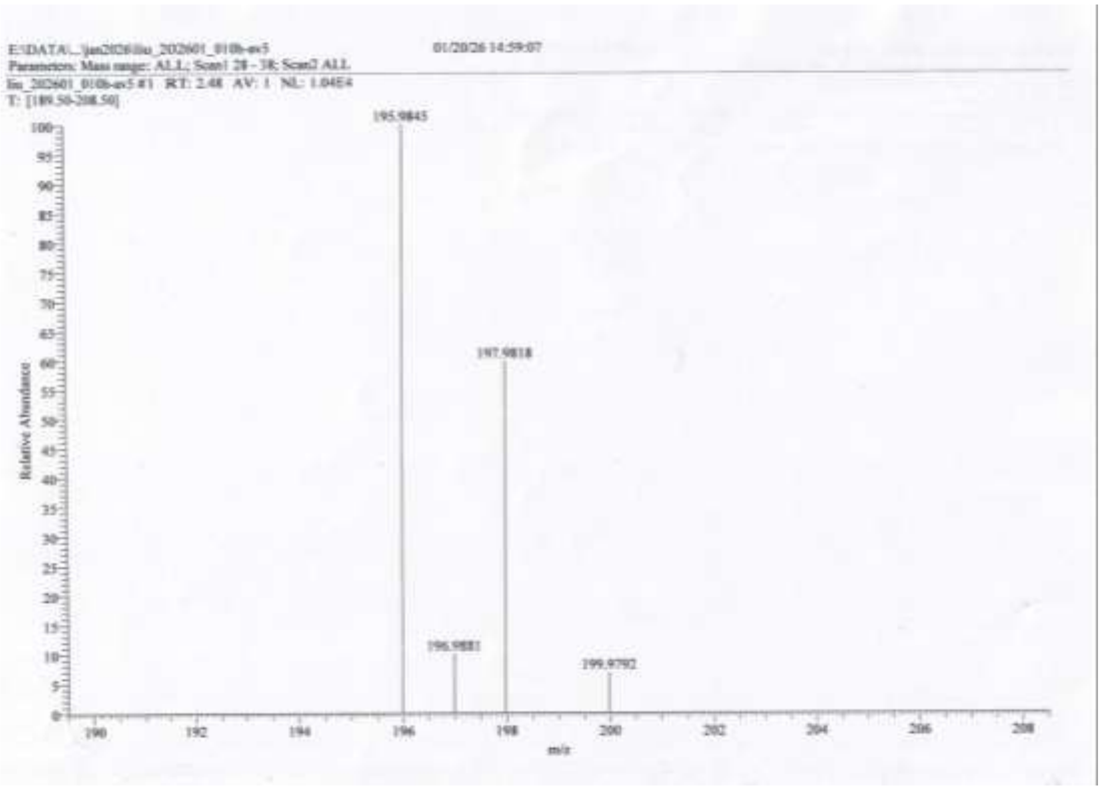

Figure S36. HR-MS (EI) spectrum for **2**.

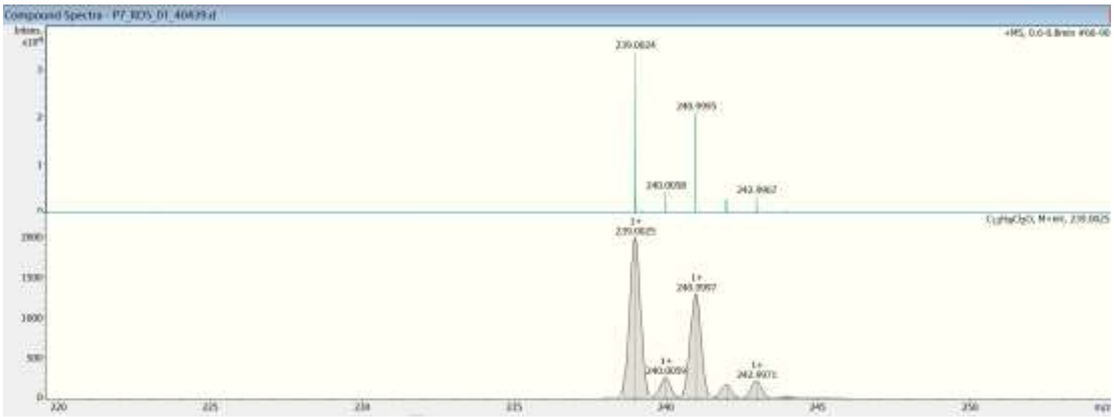

Figure S37. HR-MS (ESI) spectra for **3** (Green: experiment; Black: simulation).

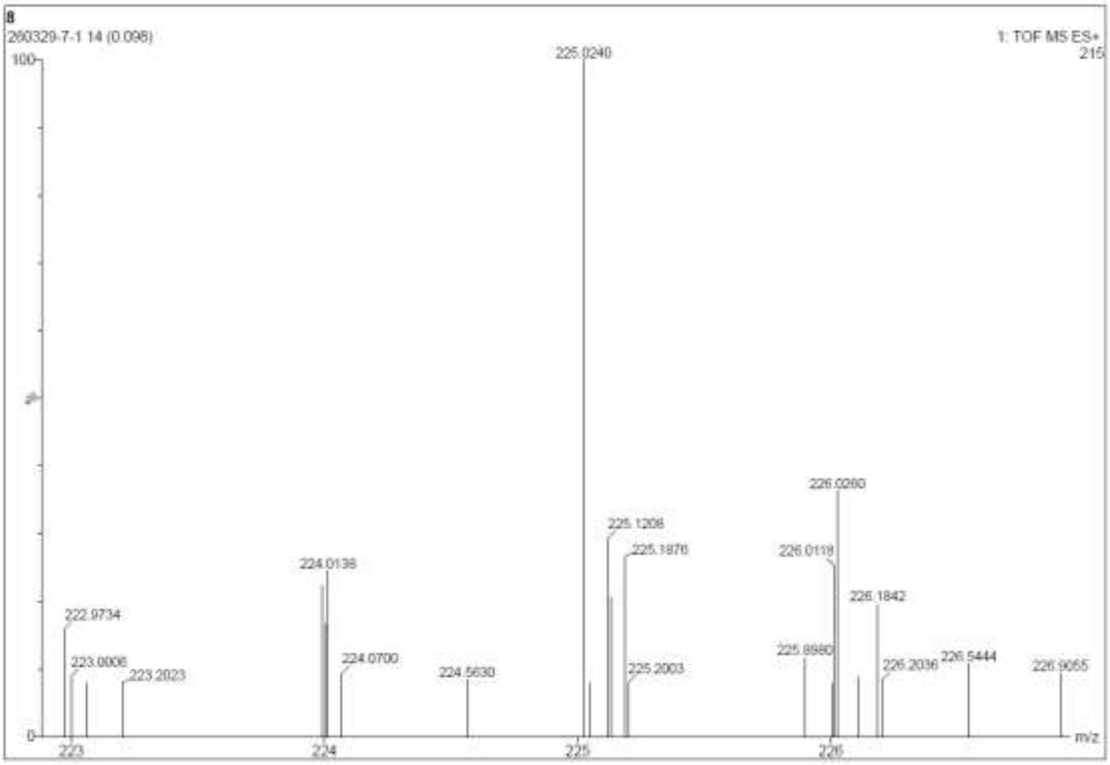

Figure S38. HR-MS spectrum of **4**.

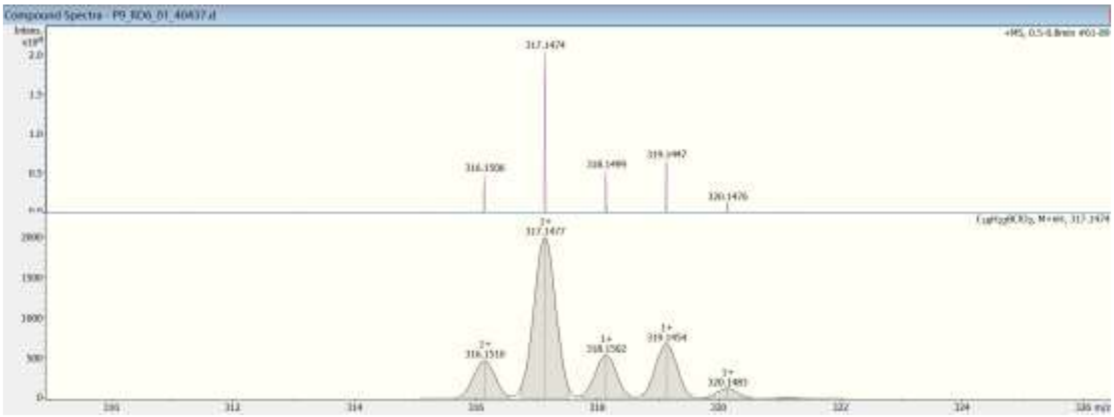

Figure S39. HR-MS (ESI) spectra for **5** (Purple: experiment; Black: simulation).

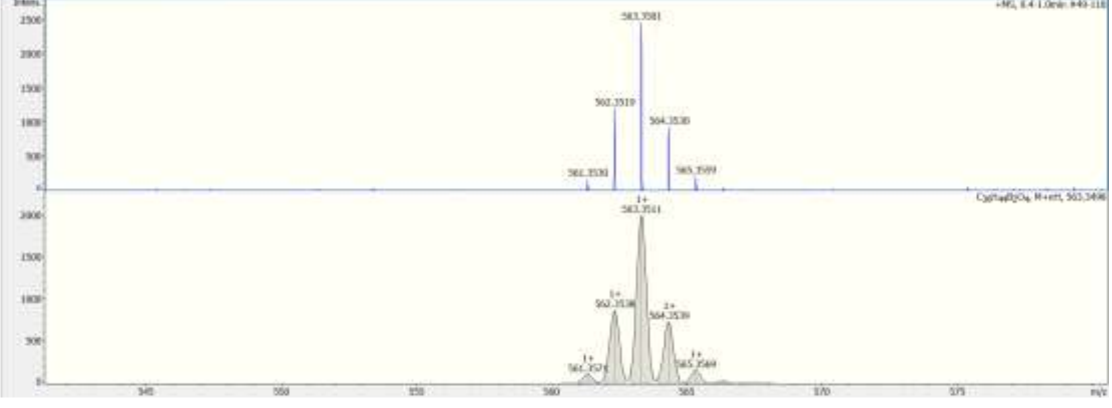

Figure S40. HR-MS (ESI) spectra for **6** (Blue: experiment; Black: simulation).

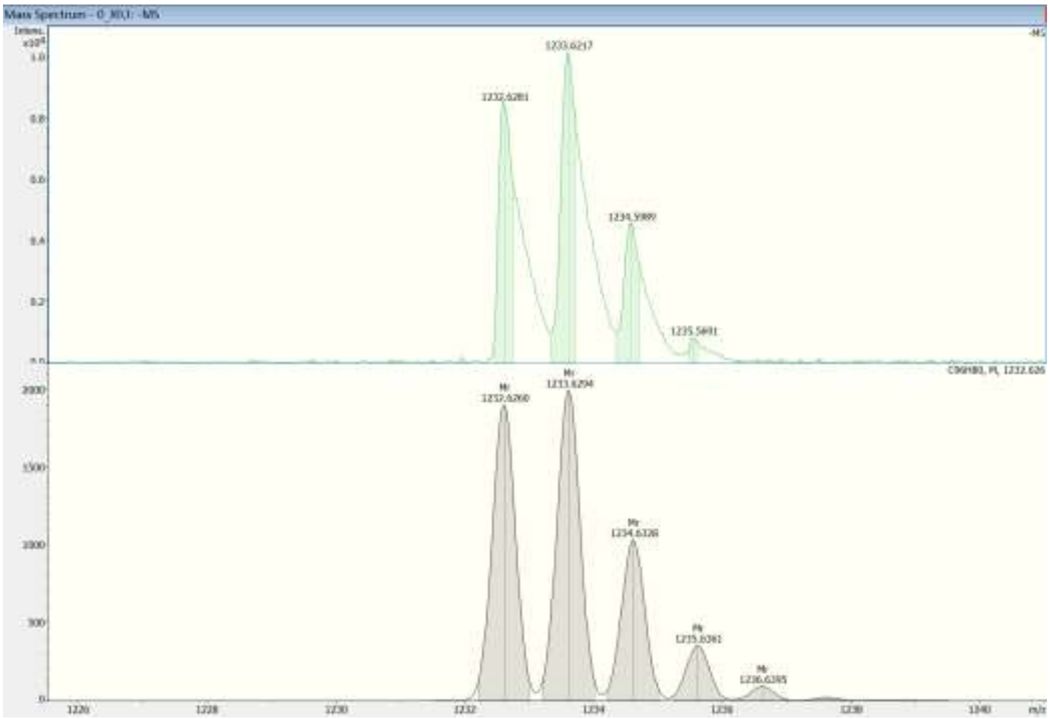

Figure S41. HR-MS (MALDI-TOF) spectra of **[8]CPA** (Green: experiment; Black: simulation).

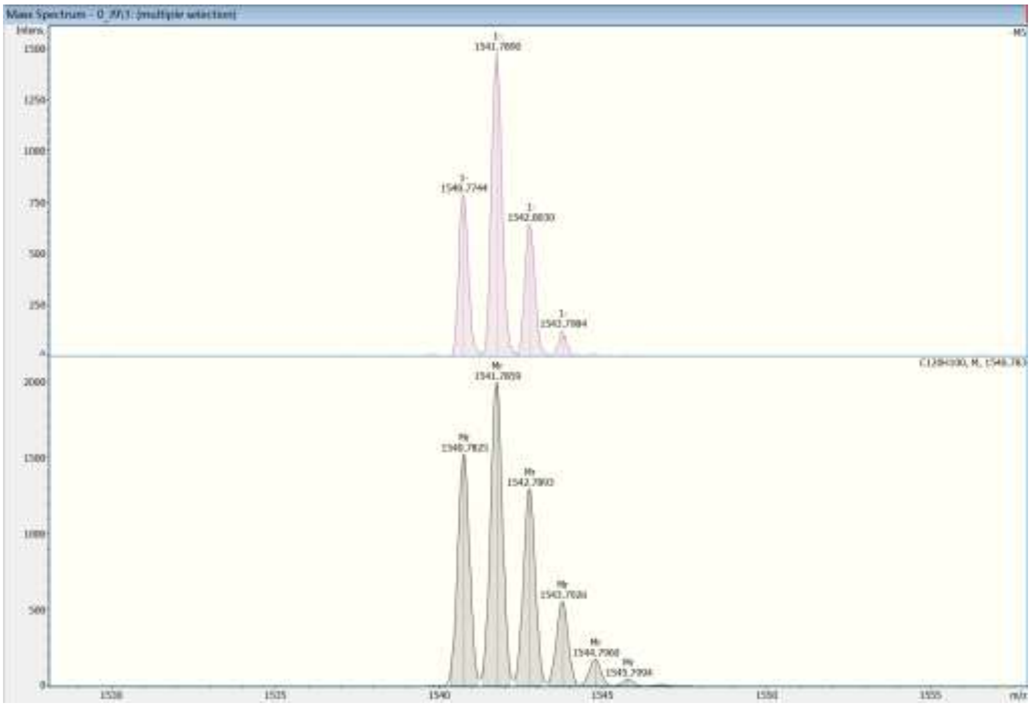

Figure S42. HR-MS (MALDI-TOF) spectra of **[10]CPA** (Purple: experiment; Black: simulation).

7. References

1. Holovics, T. C.; Robinson, R. E.; Weintrob, E. C.; Toriyama, M.; Lushington, G. H.; Barybin, M. V., The 2,6-Diisocyanoazulene Motif: Synthesis and Efficient Mono- and Heterobimetallic Complexation with Controlled Orientation of the Azulenic Dipole. *J. Am. Chem. Soc.* **2006,** *128*, 2300-2309.
2. Suzuki, Y.; Steinhart, M.; Graf, R.; Butt, H.-J.; Floudas, G., Dynamics of Ice/Water Confined in Nanoporous Alumina. *J. Phys. Chem. B.* **2015,** *119*, 14814-14820.
3. Frisch, M. J.; Trucks, G. W.; Schlegel, H. B.; Scuseria, G. E.; Robb, M. A.; Cheeseman, J. R.; Scalmani, G.; Barone, V.; Petersson, G. A.; Nakatsuji, H.; Li, X.; Caricato, M.; Marenich, A. V.; Bloino, J.; Janesko, B. G.; Gomperts, R.; Mennucci, B.; Hratchian, H. P.; Ortiz, J. V.; Izmaylov, A. F.; Sonnenberg, J. L.; Williams; Ding, F.; Lipparini, F.; Egidi, F.; Goings, J.; Peng, B.; Petrone, A.; Henderson, T.; Ranasinghe, D.; Zakrzewski, V. G.; Gao, J.; Rega, N.; Zheng, G.; Liang, W.; Hada, M.; Ehara, M.; Toyota, K.; Fukuda, R.; Hasegawa, J.; Ishida, M.; Nakajima, T.; Honda, Y.; Kitao, O.; Nakai, H.; Vreven, T.; Throssell, K.; Montgomery Jr., J. A.; Peralta, J. E.; Ogliaro, F.; Bearpark, M. J.; Heyd, J. J.; Brothers, E. N.; Kudin, K. N.; Staroverov, V. N.; Keith, T. A.; Kobayashi, R.; Normand, J.; Raghavachari, K.; Rendell, A. P.; Burant, J. C.; Iyengar, S. S.; Tomasi, J.; Cossi, M.; Millam, J. M.; Klene, M.; Adamo, C.; Cammi, R.; Ochterski, J. W.; Martin, R. L.; Morokuma, K.; Farkas, O.; Foresman, J. B.; Fox, D. J. *Gaussian 16 Rev. C.01*, Wallingford, CT, 2016.
4. Colwell, C. E.; Price, T. W.; Stauch, T.; Jasti, R., Strain visualization for strained macrocycles. *Chem. Sci.* **2020,** *11*, 3923-3930.
5. Chen, Z.; Wannere, C. S.; Corminboeuf, C.; Puchta, R.; Schleyer, P. v. R., Nucleus-Independent Chemical Shifts (NICS) as an Aromaticity Criterion. *Chem. Rev.* **2005,** *105*, 3842-3888.
6. Schleyer, P. v. R.; Maerker, C.; Dransfeld, A.; Jiao, H.; van Eikema Hommes, N. J., Nucleus-independent chemical shifts: a simple and efficient aromaticity probe. *J. Am. Chem. Soc.* **1996,** *118*, 6317-6318.
7. Wolinski, K.; Hinton, J. F.; Pulay, P., Efficient implementation of the gauge-independent atomic orbital method for NMR chemical shift calculations. *J. Am. Chem. Soc.* **1990,** *112*, 8251-8260.
8. Wang, Z., py. Aroma: An Intuitive Graphical User Interface for Diverse Aromaticity Analyses. *Chemistry.* **2024,** *6*, 1692-1703.
9. Lu, T.; Chen, F., Multiwfn: A multifunctional wavefunction analyzer. *J. Comput. Chem.* **2012,** *33*, 580-592.
10. William, H., VMD-visual molecular dynamics. *J. Mol. Graph.* **1996,** *14*, 33-38.
